# Supplementary material for: Pre-equilibrium biosensors as an approach towards rapid and continuous molecular measurements
Source: Nat Commun. 2022 Nov 18;13:7072. doi: 10.1038/s41467-022-34778-5 (PMC9674706; doi:10.1038/s41467-022-34778-5)
Supplement: Supplementary file 1 — Supplementary Information [file 41467_2022_34778_MOESM1_ESM.pdf]

Supplementary Information

Supplementary Note 1: Analysis of Mass Transport and Poisson Noise Limitations on Sensor Design

Beyond receptor properties, analyte transport to the sensor surface is an important consideration in examining the kinetics of biosensors. For our proposed pre-equilibrium model, we assume that the underlying sensor operates in a reaction-limited regime – that is, the overall sensor response is limited by the kinetics of the receptor rather than by diffusive and advective analyte transport to the sensor surface. In this reaction-limited regime, analyte transport has a negligible effect on the dynamics of the sensor response, which will then be solely governed by the law of mass-action described in Eq. 2 and 3 (Main Text). Fortunately, there are established methods for designing biosensors to ensure they operate in this regime; thus, our approach can be applied in a broad range of applications as long as these methods are considered.

Squires et al.[1] have provided a comprehensive framework for analyzing the interplay between analyte transport and reaction in surface-based biosensors, along with design principles based on this analysis. They describe the Damkohler number, Da, that determines if a sensor with given properties (e.g. geometry, sample flow-rates, surface and receptor preparation) acts within a reaction-limited or transport-limited regime. If Da $\gg$ 1, the sensor acts in the transport-limited regime, whereas if Da $\ll$ 1 the sensor is reaction-limited and thus in the regime where our analysis holds. Da is computed as

$$\mathrm{Da} = \frac{\mathrm{k_{on}} b_m L}{D\,\mathcal{F}} \tag{1}$$

where $\mathrm{k_{on}}$ is the receptor on-rate, b_m is the density of receptors on the sensor surface, and D is the diffusivity of the analyte. This model assumes the biosensor takes the form of a planar surface exposed to continuous flow of sample inside a measurement chamber with a rectangular cross-section, where L is the length of the sensor and $\mathcal{F}$ is the dimensionless flux (which is computed based on the sensor length and width, sensing channel height and width, and sample flow-rate). Using this framework, it is straightforward to design sensors that operate in a reaction-limited regime, such that our pre-equilibrium estimation approach can be applied.

As an example of this design method, we consider a microsensor of dimensions 50 x 50 μm that is supplied with sample at a flow rate of 100 μL/min – a reasonable flow-rate for human biofluids such as blood. For a representative sensor based on our proposed optimized insulin-sensing system (Supplementary Table 1), we find that Da = 0.129, and thus the sensor operates very close to the purely reaction-limited regime (Da << 1). The ratio between the overall time constant of sensor equilibration (τ_{CRD}), which incorporates effects of convection, reaction, and diffusion, and time constant of the reaction alone (τ_R,) will be bounded by $\frac{\tau_{CRD}}{\tau_R} \leq 1 + \mathrm{Da} = 1.129$. In other words, we can expect no more than a 12.9% slowdown in sensor response. Importantly, these assumptions are conservative with respect to mass transport, as the model assumes a purely laminar flow. In many cases, transport to the surface could be further enhanced – for example, using chaotic mixing by herringbone microfluidic structures[2–5] – to further drive the sensor into a reaction-limited regime where analyte transport to the sensor poses no limitation to the kinetics of sensing.

Supplementary Table 1: Representative values of sensor properties used in calculation of Poisson noise and Damkohler number calculations

Sensor Parameter	Representative Value	Notes
Receptor on-rate k_{on}	10^6 $s^{-1}M^{-1}$	Value used in the insulin sensing model in Figure 8. Note this value is relatively high for an Ab and is thus conservative with respect to the reaction-limited assumption.
Receptor surface density b_m	$1 \cdot 10^{12}$ cm^{-2}	Approximate upper limit of surface density for antibody immobilization[1], as well as conventional surface density for aptamer immobilization[6].
Sensor length L	50 μm	Typical dimensions for a microsensor.
Sensor width W_s	50 μm	
Measurement channel width W_C	50 μm	

Measurement channel height<br>H	100 µm	
Diffusivity<br>D	100 µm^2 s^{-1}	Diffusivity for insulin in aqueous solution[7].
Target concentration<br>T_0	150 pM	The maximum insulin concentration from our insulin sensing model in Figure 8. The highest concentration will give the most conservative estimate for shot noise (*i.e.,* the largest shot noise amplitude).
Receptor affinity<br>K_D	3 nM	Value used in the insulin sensing model in Figure 8.

By ensuring that our sensor acts in the reaction-limited regime, we also enforce our assumption that solution-phase analyte is in great excess relative to receptors on the sensor surface. When Da $\ll$ 1, the flux of analyte to the sensor surface through advection and diffusion is much faster than the rate of binding, offsetting any local depletion of target caused by sensor binding. As long as there is sufficient sample volume to maintain continuous flow across the sample surface, receptors on the surface will never deplete the solution, and analytes will always remain in vast excess. For example, in the scenario described above, the number of targets found within just ~13 nL of solution containing 150 pM of analyte will be sufficient to occupy the bound fraction of receptors present on the sensor surface. Thus, equilibration without depletion can be readily achieved with the 100 µL/min flow-rate utilized in the model. For continuous sensing in human biofluids such as blood, where sampling milliliter volumes is possible, these continuous flow-rates are readily attainable.

Another fundamental limitation on sensor design is imposed by the presence of Poisson noise (also termed shot noise) due to statistical fluctuations in the number of bound receptors on the sensor surface. At a given target concentration, the number of bound receptors on a sensor surface follows a Poisson distribution, where the mean number of bound receptors, $\overline{N_{bound}}$, is given by the Langmuir isotherm as

$$\overline{N_{bound}} = \frac{T_0}{T_0 + K_D} \cdot N_{total} = \frac{T_0}{T_0 + K_D} \cdot b_m L W_s \tag{2}$$

where the total number of receptors (N_{total}) can be inferred from the length (L), width (W_s) and receptor surface density (b_m) of the sensor. The standard deviation of a Poisson distribution is the square root of its mean, and thus the shot noise in terms of bound receptors is $N_{shot} = \sqrt{N_{bound}}$.

To determine if this additional Poisson noise will act as a significant factor in our system, we need to compare its amplitude to the amplitude of the zero-mean white noise that we assume to be the dominant noise in our model. This white noise has a standard deviation of $\sigma_N = 5 \cdot 10^{-3}$ fraction bound, and thus to make a comparison, we calculate the standard deviation of our Poisson distribution in terms of fraction bound as:

$$\sigma_{shot} = \frac{N_{shot}}{N_{total}} = \frac{\sqrt{\frac{T_0}{T_0 + K_D} \cdot b_m L W_s}}{b_m L W_s} = \sqrt{\frac{T_0}{(T_0 + K_D) b_m L W_s}} \quad (3)$$

This equation indicates that larger or denser sensors with more receptors will experience less fraction bound shot noise, as statistical fluctuations in bound receptors become less significant. Applying this calculation to the representative microsensor parametrized in our analysis of analyte transport to the sensor surface, we find the shot noise to be $\sigma_{shot} = 4.4 \cdot 10^{-5}$. This is much smaller than our assumed white noise ($\sigma_N = 5 \cdot 10^{-3}$), and thus can be assumed to be negligible in our analysis.

Supplementary Note 2: BLI experiment and characterization

For the characterization of antibody kinetics, a Sartorius Octet Red384 was used with anti-mouse Fc-capture biosensor tips (Sartorius). The tips were rehydrated in deionized (DI) water for 10 minutes. Anti-TNFα clones mAb1 and mAb11 were obtained from eBioscience. The antibodies were diluted to 90 nM in wash buffer (1X PBS, 0.1% Tween 20). The BLI sensor tips were dipped in antibody solution until the BLI shift was around 0.8 nm (about 60 s). The tips were then dipped in wash buffer for 180 s. Different sensor tips were then dipped in dilutions of 80 nM, 60 nM, 40 nM, 30 nM, 20 nM, and 0 nM recombinant TNFα (R&D Systems). The association curve was captured for 300 s, then the tips were moved to blank solution and the dissociation curve was observed for 900 s. ForteBio Octet DataAnalysis software was used to align and fit the data (Supplementary Figure 1a,b). The off-rate (k_{off}) was determined as the average of the k_{off} values of each dissociation trace. The fitted association rates (k_{obs}) were then plotted vs target concentration (Supplementary Figure 1c). The on-rate (k_{on}) was determined as the slope of the best fit line, $k_{obs} = k_{on}[T] + k_{off}$.

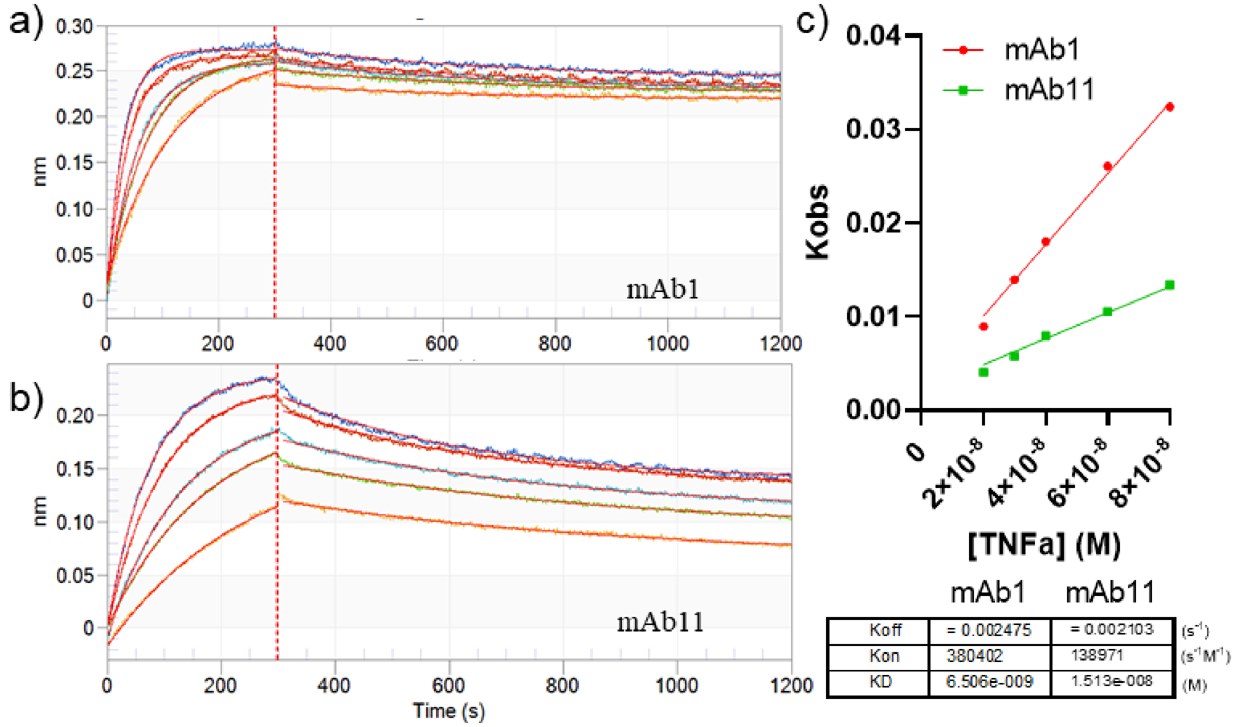

	mAb1	mAb11	
Koff	= 0.002475	= 0.002103	(s^{-1})
Kon	380402	138971	($s^{-1}M^{-1}$)
KD	6.506e-009	1.513e-008	(M)

Supplementary Figure 1: BLI Characterization. a) mAb1 and b) mAb11 association and dissociation data, fitted with the ForteBio Data Analysis software. c) Fit of k_{obs} vs target concentration used to determine k_{on}.

The data for the 20 nM experiment was truncated at 600 seconds and normalized by dividing all measurements by the RMax value provided by the analysis software (0.31654 nm for mAb1, 0.29594 nm for mAb11. This is the BLI shift value corresponding to a fraction bound equal to 1) after subtracting the baseline (0.004509 for mAb1, -0.01805 for mAb11). These normalized data are shown in Figure 3a and were used to produce the results in Figure 3b and c. The normalized data were downsampled to 1/15 Hz and digitally low pass filtered with a sharp cutoff at $f_T = 0.03$ Hz. The pre-equilibrium TEA was then applied, as described by Eq. 7 (Main Text).

The equilibrium estimates shown in Figure 3b and c were calculated as follows. The equilibrium BLI shift values of the fitted association and dissociation curves were taken from the analysis software, normalized via RMax and baseline as described above to obtain equilibrium fraction bound values, and then the inverse-Langmuir was applied to compute the corresponding equilibrium concentration estimates. These values are shown in Supplementary Table 2.

Supplementary Table 2: Calculation of target estimates based on BLI equilibrium fraction bound.

	mAb1	mAb11
BLI Shift at equilibrium<br>20 nM - association	0.2613 nm	0.1508 nm
Fraction bound at equilibrium<br>20 nM - association	0.8112	0.5706
Inverse-Langmuir at equilibrium<br>20 nM - association	28 nM	20 nM
BLI Shift at equilibrium<br>0 nM - dissociation	0.2185	0.07245
Fraction bound at equilibrium<br>0 nM - dissociation	0.6760	0.3058
Inverse-Langmuir at equilibrium<br>0 nM - dissociation	13.5 nM	7 nM

A similar procedure was followed for the remaining data of Supplementary Figure 1b and is included in Supplementary Figure 2 as additional proof-of-principle data. In this figure, we provide a concentration-correlation curve (Supplementary Figure 2c) to compare the actual TNFα concentrations in the first binding step to the concentrations predicted by the inverse-Langmuir and the TEA at the timepoints 15 s, 100 s, and 200 s. This data shows that the TEA estimates the target concentration instantaneously (less than 15s), while the inverse-Langmuir, due to slow receptor equilibration, remains highly inaccurate even 200 s after exposure to the target. Supplementary Figure 2d shows the concentrations predicted by the inverse-Langmuir and the

TEA while the sensor was in blank solution, at the timepoints 400 s and 600 s. The figure also includes estimates produced by the inverse-Langmuir after very long equilibration times. The data shows that the TEA estimates concentrations that are close to the equilibrium value of the sensor nearly instantaneously.

Supplementary Table 3: Calculation of target estimates based on BLI equilibrium fraction bound for f Supplementary Figure 2.

Actual concentration	20 nM	30 nM	40 nM	60 nM	80 nM
RMax (nm)	0.29594				
Baseline (nm)	-0.01805	-0.001899	-0.006676	4.652e-005	-0.0001254
BLI Shift at equilibrium - association	0.1508 nm	0.1941 nm	0.2074 nm	0.2307 nm	0.2403 nm
Fraction bound at equilibrium - association	0.5706	0.6623	0.7234	0.7794	0.8124
Inverse-Langmuir at equilibrium - association	20 nM	30 nM	40 nM	53 nM	65.5 nM
BLI Shift at equilibrium 0 nM - dissociation	0.07245 nm	0.09743 nm	0.1098 nm	0.1265 nm	0.1291 nm
Fraction bound at equilibrium 0 nM - dissociation	0.3058	0.3356	0.3936	0.4273	0.4367
Inverse-Langmuir at equilibrium 0 nM - dissociation	7 nM	8 nM	10 nM	11 nM	12 nM

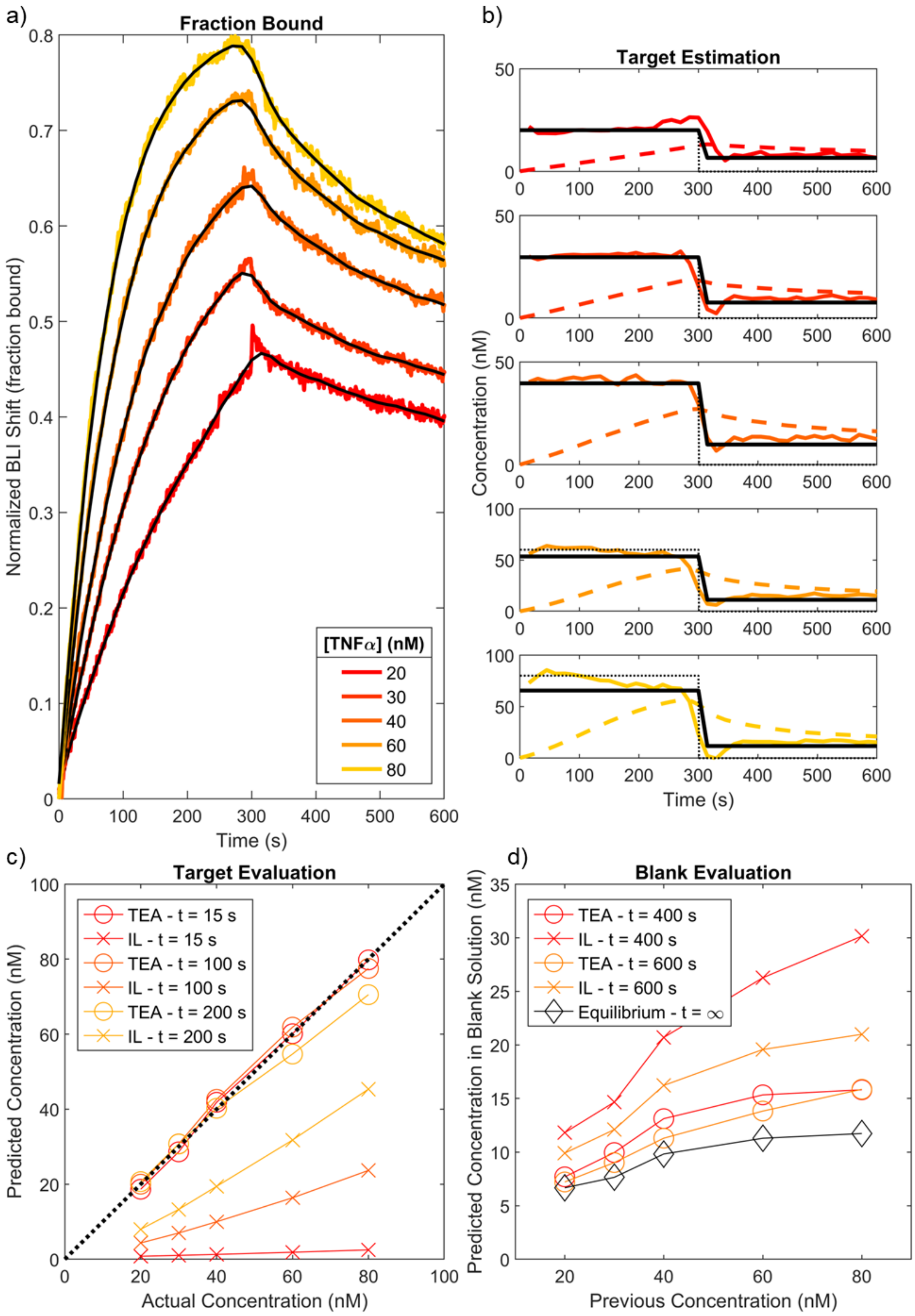

a)
Fraction Bound
Normalized BLI Shift (fraction bound)
Time (s)
[TNFα] (nM)
20
30
40
60
80
b)
Target Estimation
Concentration (nM)
Time (s)
c)
Target Evaluation
Predicted Concentration (nM)
Actual Concentration (nM)
TEA - t = 15 s
IL - t = 15 s
TEA - t = 100 s
IL - t = 100 s
TEA - t = 200 s
IL - t = 200 s
d)
Blank Evaluation
Predicted Concentration in Blank Solution (nM)
Previous Concentration (nM)
TEA - t = 400 s
IL - t = 400 s
TEA - t = 600 s
IL - t = 600 s
Equilibrium - t = ∞

Supplementary Figure 2: Additional proof-of-principle data. a) Normalized biolayer interferometry (BLI) binding data using mAb11. From 0–300 s the probe was exposed to different TNFα concentrations; from 300–600 s, the probe was dipped in blank solution. Black lines represent digitally low-pass filtered data. b) The filtered data was used to estimate the target concentration using the pre-equilibrium TEA (solid lines) as well as the conventional inverse-Langmuir (dashed lines). Solid black lines represent the inverse-Langmuir target estimates based on the fraction of bound receptors at equilibrium. Dotted black lines represent the true target concentration in solution. c) The target estimates at t = 15 s, 100 s and 200 s for both TEA and inverse Langmuir were extracted and plotted against the actual target concentrations in solution. Dotted black line corresponds to equality between predicted and actual concentrations. d) TEA and inverse-Langmuir target estimates at t = 400s and 600s, which correspond to when all BLI probes were in blank solution, were extracted and plotted against the concentration to which the probes were exposed prior to the blank sample. Black diamonds show the predicted concentration if the sensor reached equilibrium.

Supplementary Note 3: Derivation of the Frequency Response of the Receptor

Consider a bimolecular sensor where U stands for unbound receptor and B is bound receptor that generates the reporting signal.

$$U + T \rightleftharpoons B$$

The observable sensor response is $y(t) = [B]$. The target signal is $T(t)$, a time-varying concentration, $[T]$, of target. The differential equation that describes this system is:

$$\frac{dy(t)}{dt} = -k_{off}y(t) + k_{on}\big(1 - y(t)\big)T(t). \tag{4}$$

Taking the Fourier transform of the system equation, assuming no initial conditions, we obtain:

$$j2\pi fY(f) = -k_{off}Y(f) - k_{on}T(f) * Y(f) + T(f)k_{on}, \tag{5}$$

where $Y(f)$ and $T(f)$ represent the Fourier transforms of the sensor response and target signal, respectively, and $*$ is the convolution operator. Specifically, we look at the case where $T(t)$ is a sinusoidal input of one frequency f_{T}, plus an offset term. This leads to

$$T(f) = \frac{T_1}{2}\big(\delta(f - f_{\mathrm{T}}) + \delta(f + f_{\mathrm{T}})\big) + T_0\delta(f). \tag{6}$$

Using this expression in the convolution of Eq. 5 leads to:

$$T(f) * Y(f) = \int_{-\infty}^{\infty}\left[\frac{T_1}{2}\big(\delta(f - f_{\mathrm{T}}) + \delta(f + f_{\mathrm{T}})\big) + T_0\delta(\omega)\right]Y(f - f_1)df_1 \tag{7}$$

which simplifies to:

$$T(f) * Y(f) = T_0Y(f) + \frac{T_1}{2}\big(Y(f - f_{\mathrm{T}}) + Y(f + f_{\mathrm{T}})\big). \tag{8}$$

Returning to the Fourier transform of the system, Eq. 5, combining it with Eq. 6 and 8, and simplifying, we obtain:

$$Y(f) = \alpha\left[\frac{T_1}{2}\big(\delta(f - f_{\mathrm{T}}) + \delta(f + f_{\mathrm{T}})\big) + T_0\delta(f)\right] - \frac{T_1}{2}\alpha\big(Y(f - f_{\mathrm{T}}) + Y(f + f_{\mathrm{T}})\big), \tag{9}$$

where $\alpha = \frac{k_{on}}{k_{off}+k_{on}C_0+j2\pi f}$. We then assume that the fraction bound signal produced by the receptor is also sinusoidal, with a frequency equal to that of the target. Thus, analogously to Eq. 6, we can write

$$Y(f) = \frac{C_1}{2}\left(\delta(f - f_{\mathrm{T}}) + \delta(f + f_{\mathrm{T}})\right) + C_0\delta(f). \tag{10}$$

Combining Eq. 9 and 10, we obtain an expression with multiple frequency terms, whose amplitude factors depend on α, T_0, T_1, C_0, and C_1. We apply harmonic balance equating all terms of the same frequency for the terms corresponding to $\delta(f)$, $\delta(f + f_T)$ and $\delta(f - f_T)$. Doing so, we obtain a system of two equations:

$$\text{For } \delta(f)\text{:} \quad C_0 = \alpha T_0 - \frac{\alpha T_1}{2} C_1, \tag{11}$$

$$\text{For } [\delta(f + f_T) + \delta(f - f_T)]\text{:} \quad C_1 = \alpha T_1 (1 - C_0). \tag{12}$$

We solve these equations first by setting $f = 0$ for C_0, as the harmonic corresponding to this amplitude term has zero frequency. Doing so, we obtain:

$$C_0 = \frac{2(K_D + T_0)T_0 - T_1^2}{2(K_D + T_0)^2 - T_1^2}. \tag{13}$$

Assuming that the amplitude of oscillation is smaller than the mean concentration, $T_1 < T_0$, then Eq. 13 reduces to:

$$C_0 = \frac{\frac{T_0}{K_D}}{1 + \frac{T_0}{K_D}} = \frac{k_{on}T_0}{k_{off} + k_{on}T_0}, \tag{14}$$

which is the Langmuir isotherm. This indicates that the harmonic balance analysis correctly predicts the behavior of the receptor responding to a zero-frequency target concentration. Next, solving the system for the transfer function C_1/T_1 and setting $f = f_T$, we obtain:

$$\frac{C_1}{T_1} = \frac{k_{on}}{k_{off} + T_0 k_{on}} \cdot \frac{k_{off}}{k_{off} + k_{on}T_0 + j2\pi f_T} \approx \frac{k_{on}}{k_{off} + T_0 k_{on}} \cdot \frac{1}{1 + j\frac{2\pi f_{\mathrm{T}}}{k_{off} + k_{on}T_0}}, \tag{15}$$

where the approximation step assumes that $K_D > T_0$, such that $k_{on}T_0 < k_{off}$. This allows us to massage the equation into the form of a quasi-Langmuir multiplied by a first-order low-pass filter.

Supplementary Note 4: Derivation of Frequency Response of TEA

Re-arranging the law of mass action and solving for the target concentration signal yields:

$$T(t) = \frac{\frac{dy(t)}{dt} + k_{off}y(t)}{k_{on}\big(1 - y(t)\big)}. \tag{16}$$

As mentioned in the main text, for the case of the model system that we analyzed, this equation is discretized to describe the operation of the TEA. This leads to the expression,

$$E[n] = \frac{w'[n] + k_{off}w[n]}{k_{on}(1 - w[n])}, \tag{17}$$

where $E[n]$ now represents the reconstructed estimate of the target concentration. If the sampling frequency of the sensor is large compared to the frequency content of the target signal, we can estimate the derivative term $w'[n]$ at any index n as:

$$w'[n] = (w[n] - w[n-1])f_S. \tag{18}$$

Combining Eq. 17 and 18 and taking the discrete-time Fourier transform results in the expression:

$$W\left[e^{j\omega}\right]\left(k_{off} + f_S\left(1 - e^{-j\omega}\right)\right) = k_{on}E\left[e^{j\omega}\right] - k_{on}E\left[e^{j\omega}\right] * W\left[e^{j\omega}\right], \tag{19}$$

where j is the imaginary unit, ω is the normalized discrete time fourier transform frequency, $\omega = 2\pi f/f_S$, and $*$ represents the circular convolution of the 2π-periodic DTFTs of E and W given by:

$$E\left[e^{j\omega}\right] * W\left[e^{j\omega}\right] = \frac{1}{2\pi}\int_{-\pi}^{\pi} W\left[e^{j\omega_1}\right] \cdot E\left[e^{j(\omega-\omega_1)}\right]d\omega_1. \tag{20}$$

If we now assume that $w[n]$ are samples of $C_1\cos(2\pi f_T t) + C_0$ and $\Omega_T = 2\pi f_T/f_S$, then we can write the DTFT of $w[n]$ as:

$$W\left[e^{j\omega}\right] = \pi\sum_{k=-\infty}^{\infty} 2C_0\delta(\omega - 2\pi k) + C_1\delta(\omega - \Omega_T - 2\pi k) + C_1\delta(\omega + \Omega_T - 2\pi k). \tag{21}$$

The circular convolution, Eq. 20, then becomes:

$$E[e^{j\omega}] * W[e^{j\omega}] = C_0 \int_{-\pi}^{\pi} \delta(\omega_1) E[e^{j(\omega-\omega_1)}] d\omega_1 + \frac{1}{2} C_1 \int_{-\pi}^{\pi} \delta(\omega_1 - \Omega_T) E[e^{j(\omega-\omega_1)}] \, d\omega_1 + \frac{1}{2} C_1 \int_{-\pi}^{\pi} \delta(\omega_1 + \Omega_T) E[e^{j(\omega-\omega_1)}] \, d\omega_1, \tag{22}$$

Which simplifies to:

$$E[e^{j\omega}] * W[e^{j\omega}] = C_0 E[e^{j\omega}] + \frac{1}{2} C_1 E[e^{j(\omega-\Omega_C)}] + \frac{1}{2} C_1 E[e^{j(\omega+\Omega_C)}]. \tag{23}$$

Inserting Eq. 21 and 23 into Eq. 19 and simplifying, we obtain:

$$\alpha \sum_k [2C_0 \delta(\omega - 2\pi k) + C_1(\delta(\omega - \Omega_T - 2\pi k) + \delta(\omega + \Omega_T - 2\pi k))] + \beta(E[e^{j(\omega-\Omega_C)}] + E[e^{j(\omega+\Omega_C)}]) = E[e^{j\omega}] \tag{24}$$

where $\alpha = \frac{\pi(k_{off} + (1-e^{-j\omega}) f_S)}{k_{on}(1-C_0)}$ and $\beta = \frac{C_1}{2(1-C_0)}$. Next, we assume that the output signal $E[n]$ will also take the form of a sinusoid of the same frequency as the input, and thus has DTFT of the form:

$$E[e^{j\omega}] = \pi \sum_{k=-\infty}^{\infty} 2D_0 \delta(\omega - 2\pi k) + D_1 \delta(\omega - \Omega_T - 2\pi k) + D_1 \delta(\omega + \Omega_T - 2\pi k). \tag{25}$$

Combining Eq. 24 and 25, we obtain an equation of 2π-periodic DTFTs with multiple frequency components. The amplitude factors corresponding to these sinusoids depend on α, β, C_0, C_1, D_0, and D_1. We apply the harmonic balance technique, considering only the fundamental frequency terms $\delta(\omega)$, $\delta(\omega + \Omega_T)$ and $\delta(\omega - \Omega_T)$, and equating all terms of the same frequency, we obtain the following system of equations:

$$\text{For } \delta(\omega): \quad \frac{2\alpha}{\pi} C_0 + 2\beta D_1 = 2D_0, \tag{26}$$

$$\text{For } [\delta(\omega + \Omega_T) + \delta(\omega - \Omega_T)]: \quad \frac{\alpha}{\pi} C_1 + 2\beta D_0 = D_1. \tag{27}$$

Solving these two equations for D_0 first, we set $\omega = 0$, as the harmonic corresponding to this term has zero frequency. Doing so, we obtain:

$$D_0 = K_D \cdot \frac{(1 - C_0)C_0 + C_1^2/2}{(1 - C_0)^2 - C_1^2/2}. \tag{28}$$

Assuming that the amplitude of oscillation is smaller than the mean concentration, $C_1 < C_0$, then Eq. 28 reduces to:

$$D_0 = K_D \cdot \frac{C_0}{1 - C_0}. \quad (29)$$

Eq. 29 is the inverse-Langmuir, indicating that the analysis correctly predicts that a constant fraction bound with value C_0 at the input of the TEA will be transformed into an estimated constant concentration D_0 by inverting the equilibrium behavior of the receptor.

Next, solving the system for the transfer function D_1/C_1, and setting $\omega = \Omega_T$, we obtain:

$$\frac{D_1}{C_1} = \frac{1}{1 - C_0} \cdot \frac{f_S}{k_{on}} \left(\frac{k_{off}}{f_S} \left(1 + \frac{C_0}{1 - C_0} \right) + 1 - e^{-j\Omega_T} \right). \quad (30)$$

We can greatly simplify this equation by assuming that $C_0 \ll 1$; *i.e.*, assuming that the sensor will likely be operating in a regime where $K_D > T_0$. Taking this assumption reduces Eq. 30 to:

$$\frac{D_1}{C_1} = \frac{1}{1 - C_0} K_D \left(1 + \frac{f_S}{k_{off}} \left(1 - e^{-j\Omega_T} \right) \right). \quad (31)$$

Subsequent analyses conducted in this paper can also be carried out with the more accurate expression of Eq. 30. However, if the intent is to analyze a sensor operating in the regime where $K_D \ll T_0$, one must consider that the strong nonlinearities of the isotherm close to saturation will affect the accuracy of the analytical models presented in this discussion.

Supplementary Note 5: Noise Scale Function

The average noise power at the output of the TEA, $\overline{\sigma^2_{N,Out}}$, is given by the sum of output power spectral density at all frequencies. The square of the frequency response of the TEA multiplied by the power spectral density at the input of the TEA gives the output power spectral density. However, we assume that digital filtering and averaging are being used, such that all frequency content above the highest target frequency of interest, f_T, is removed. Thus, the average noise power is given by:

$$\overline{\sigma^2_{N,Out}}(f_T, f_S) = \frac{1}{2\pi}\int_{-f_T}^{f_T} \frac{N_0}{2}\left|\frac{D_1}{C_1}(f, f_S)\right|^2 df, \quad (32)$$

and the root mean square (RMS) noise, in units of moles$_{\mathrm{RMS}}$, in the target estimate $E[n]$ is given by $RMS_N = \sqrt{\overline{\sigma^2_{N,Out}}}$. It is useful to re-write Eq. 32 as:

$$\overline{\sigma^2_{N,Out}} = \frac{N_0}{2} \cdot S_N(f_T, f_S),$$

where the noise scaling function $S_N(f_T, f_S)$ with units of Hz $\left(\frac{\text{moles}}{\text{fraction bound}}\right)^2$ isolates the impact of the TEA. If we plug in:

$$\frac{D_1}{C_1} = K_D \cdot \frac{1}{1 - C_0}\left(1 + \frac{f_S}{k_{off}}\left(1 - e^{-j\frac{2\pi f_T}{f_S}}\right)\right) = (K_D + T_0)\left(1 + \frac{f_S}{k_{off}}\left(1 - e^{-j\frac{2\pi f_T}{f_S}}\right)\right) \quad (33)$$

and evaluate the integral using Wolfram Mathematica, we obtain:

$$S_N(f_T, f_S) = (K_D + T_0)^2\left(f_T\left(\frac{2f_S^2}{k_{off}^2} + \frac{2f_S}{k_{off}} + 1\right) - \frac{f_S^2}{\pi}\left(\frac{f_S}{k_{off}^2} + \frac{1}{k_{off}}\right)\sin\left(\frac{2\pi f_T}{f_S}\right)\right). \quad (34)$$

This expression for the noise scaling function can be used to calculate how the TEA propagates noise to the output. Importantly, this function can be minimized in terms of the thermodynamics and kinetics of the receptor, as is described in the main text.

Supplementary Note 6: Glossary of symbols

k_{off}	Off-rate kinetic constant in units of s^{-1}.
k_{on}	On-rate kinetic constant in units of $s^{-1}M^{-1}$.
K_D	Dissociation constant in units of M.
$T(t)$	Target concentration changing as a function of time t in units of M.
$y(t)$	Receptor fraction bound response to target concentration as a function of time.
$y[n]$	Samples of $y(t)$ before noise is introduced.
$w[n]$	Noisy samples of receptor fraction bound produced by the detector.
$E[n]$	Estimate values of target concentration $T(t)$ produced by the TEA.
$N[n]$	White gaussian noise term added by detector. $w[n] = y[n] + N[n]$.
$N_0/2$	Power spectral density of $N[n]$ in units of $(\text{fraction bound})^2/\text{Hz}$.
f_S	Sampling frequency of the detector in units of Hz.
f_T	Target frequency in Hz used in harmonic balance analysis. Also, highest frequency of interest in target signal – sets cutoff of digital low-pass filter.
Ω_T	$2\pi f_T$ in units of rad.
$\Omega_{T,S}$	$2\pi f_T/f_S$ i.e. Ω_T normalized to the sampling frequency.
Ω_C	Cutoff frequency of the receptor.
T_1	Amplitude of oscillation of the target signal in units of M.
T_0	Mean target concentration in units of M.
C_1	Amplitude of oscillation of the receptor fraction bound response.
C_0	Mean of the receptor fraction bound response.
D_1	Amplitude of oscillation of the target estimate in units of M.
D_0	Mean of the target estimate in units of M.
$\overline{\sigma_{N,Out}^2}$	Average noise power in $E[n]$.
RMS_N	Root mean squared noise in $E[n]$.
$S_N(f_T, f_S)$	Noise scaling function with units of Hz $\left(\frac{\text{moles}}{\text{fraction bound}}\right)^2$ relates $N_0/2$ with $\overline{\sigma_{N,Out}^2}$.
σ_N	Detector fraction bound noise standard deviation.

Supplementary References

1. Squires, T. M., Messinger, R. J. & Manalis, S. R. Making it stick: Convection, reaction and diffusion in surface-based biosensors. *Nat Biotechnol* 26, 417–426 (2008).

2. Nguyen, N. T. & Wu, Z. Micromixers - A review. *Journal of Micromechanics and Microengineering* vol. 15 1–16 Preprint at https://doi.org/10.1088/0960-1317/15/2/R01 (2005).

3. Ottino, J. M. & Wiggins, S. Introduction: Mixing in microfluidics. *Philosophical Transactions of the Royal Society A: Mathematical, Physical and Engineering Sciences* vol. 362 923–935 Preprint at https://doi.org/10.1098/rsta.2003.1355 (2004).

4. Poudineh, M. *et al.* A fluorescence sandwich immunoassay for the real-time continuous detection of glucose and insulin in live animals. *Nat Biomed Eng* 5, 53–63 (2021).

5. Williams, M. S., Longmuir, K. J. & Yager, P. A practical guide to the staggered herringbone mixer. *Lab Chip* 8, 1121–1129 (2008).

6. Chien, J. C., Baker, S. W., Soh, H. T. & Arbabian, A. Design and Analysis of a Sample-and-Hold CMOS Electrochemical Sensor for Aptamer-Based Therapeutic Drug Monitoring. *IEEE J Solid-State Circuits* 55, 2914–2929 (2020).

7. Ling, G. N. & Kromash, M. H. The extracellular space of voluntary muscle tissues. *J Gen Physiol* 50, 677–694 (1967).
