# Supplementary material for: Pre-equilibrium biosensors as an approach towards rapid and continuous molecular measurements
Source: Nat Commun. 2022 Nov 18;13:7072. doi: 10.1038/s41467-022-34778-5 (PMC9674706; doi:10.1038/s41467-022-34778-5)
Supplement: Supplementary file 3 — Reporting Summary [file 41467_2022_34778_MOESM3_ESM.pdf]

Corresponding author(s): Tom Soh

Last updated by author(s): Jun 9, 2022

Reporting Summary

Nature Portfolio wishes to improve the reproducibility of the work that we publish. This form provides structure for consistency and transparency in reporting. For further information on Nature Portfolio policies, see our Editorial Policies and the Editorial Policy Checklist.

Statistics

For all statistical analyses, confirm that the following items are present in the figure legend, table legend, main text, or Methods section.

n/a	Confirmed	
☐	☒	The exact sample size (*n*) for each experimental group/condition, given as a discrete number and unit of measurement
☒	☐	A statement on whether measurements were taken from distinct samples or whether the same sample was measured repeatedly
☒	☐	The statistical test(s) used AND whether they are one- or two-sided<br>*Only common tests should be described solely by name; describe more complex techniques in the Methods section.*
☒	☐	A description of all covariates tested
☐	☒	A description of any assumptions or corrections, such as tests of normality and adjustment for multiple comparisons
☐	☒	A full description of the statistical parameters including central tendency (e.g. means) or other basic estimates (e.g. regression coefficient) AND variation (e.g. standard deviation) or associated estimates of uncertainty (e.g. confidence intervals)
☒	☐	For null hypothesis testing, the test statistic (e.g. F, t, r) with confidence intervals, effect sizes, degrees of freedom and P value noted<br>*Give P values as exact values whenever suitable.*
☒	☐	For Bayesian analysis, information on the choice of priors and Markov chain Monte Carlo settings
☒	☐	For hierarchical and complex designs, identification of the appropriate level for tests and full reporting of outcomes
☒	☐	Estimates of effect sizes (e.g. Cohen's d, Pearson's r), indicating how they were calculated

Our web collection on statistics for biologists contains articles on many of the points above.

Software and code

Policy information about availability of computer code

Data collection	Octet DataAquisition Version 10.0
Data analysis	Matlab R2021a, Octet DataAnalysis Version 10.0.3.1, GraphPad Prism 9.

For manuscripts utilizing custom algorithms or software that are central to the research but not yet described in published literature, software must be made available to editors and reviewers. We strongly encourage code deposition in a community repository (e.g. GitHub). See the Nature Portfolio guidelines for submitting code & software for further information.

Data

Policy information about availability of data

All manuscripts must include a data availability statement. This statement should provide the following information, where applicable:
- Accession codes, unique identifiers, or web links for publicly available datasets
- A description of any restrictions on data availability
- For clinical datasets or third party data, please ensure that the statement adheres to our policy

All data generated or analyzed during this study are included in this published article (and its supplementary information files).

Human research participants

Policy information about studies involving human research participants and Sex and Gender in Research.

Reporting on sex and gender	No human participants were involved in this manuscript.
Population characteristics	No human participants were involved in this manuscript.
Recruitment	No human participants were involved in this manuscript.
Ethics oversight	No ethics evaluation was required for this work.

Note that full information on the approval of the study protocol must also be provided in the manuscript.

Field-specific reporting

Please select the one below that is the best fit for your research. If you are not sure, read the appropriate sections before making your selection.

☒ Life sciences ☐ Behavioural & social sciences ☐ Ecological, evolutionary & environmental sciences

For a reference copy of the document with all sections, see nature.com/documents/nr-reporting-summary-flat.pdf

Life sciences study design

All studies must disclose on these points even when the disclosure is negative.

Sample size	We did not perform a sample size calculation. Most data presented in this paper is computational and therefore sample size is irrelevant. For proof-of-concept experimental data, we chose to demonstrate the algorithm operating on two different monoclonal antibodies. This is sufficient because all monoclonal antibodies bind via the same mechanism which is described by the same binding equations. Demonstrating that the algorithm works appropriately on these two clones shows that the algorithm will work on any monoclonal antibody.
Data exclusions	No data was excluded
Replication	Biolayer interferometry was carried out according to instrument manufacturer instructions with five different concentrations and one reference sensor trace. Different concentrations were used to verify that the kinetics of the antibodies were consistent across experiments.
Randomization	This is irrelevant to the study. The experimental data in this paper concerns the characterization of antibodies. Therefore, the 'samples' tested were created during the experiment by spiking in controlled amounts of target and the identity of each sample was known.
Blinding	The identity of each sample was known ahead of time, so no blinding was required.

Reporting for specific materials, systems and methods

We require information from authors about some types of materials, experimental systems and methods used in many studies. Here, indicate whether each material, system or method listed is relevant to your study. If you are not sure if a list item applies to your research, read the appropriate section before selecting a response.

Materials & experimental systems

n/a	Involved in the study	
☐	☒	Antibodies
☒	☐	Eukaryotic cell lines
☒	☐	Palaeontology and archaeology
☒	☐	Animals and other organisms
☒	☐	Clinical data
☒	☐	Dual use research of concern

Methods

n/a	Involved in the study	
☒	☐	ChIP-seq
☒	☐	Flow cytometry
☒	☐	MRI-based neuroimaging

Antibodies

Antibodies used	TNFa Mouse anti-Human Clone Name: mAb1, Supplier: Invitrogen, Catalog # 14734885;<br>TNFa Mouse anti-Human Clone Name: mAb11, Supplier: Invitrogen, Catalog # 12734982.

Validation

The antibodies were fully validated by the authors using standard biolayer interferometry (BLI) protocols. The antibodies were exposed to a range of target concentrations and the kinetics of binding were recorded. The equilibrium binding constants were computed based on this data and found to match typical values for monoclonal antibodies. All relevant data is included in the manuscript and SI.
