# Supplementary figures and images for: Pre-equilibrium biosensors as an approach towards rapid and continuous molecular measurements
Source: Nat Commun. 2022 Nov 18;13:7072. doi: 10.1038/s41467-022-34778-5 (PMC9674706; doi:10.1038/s41467-022-34778-5)

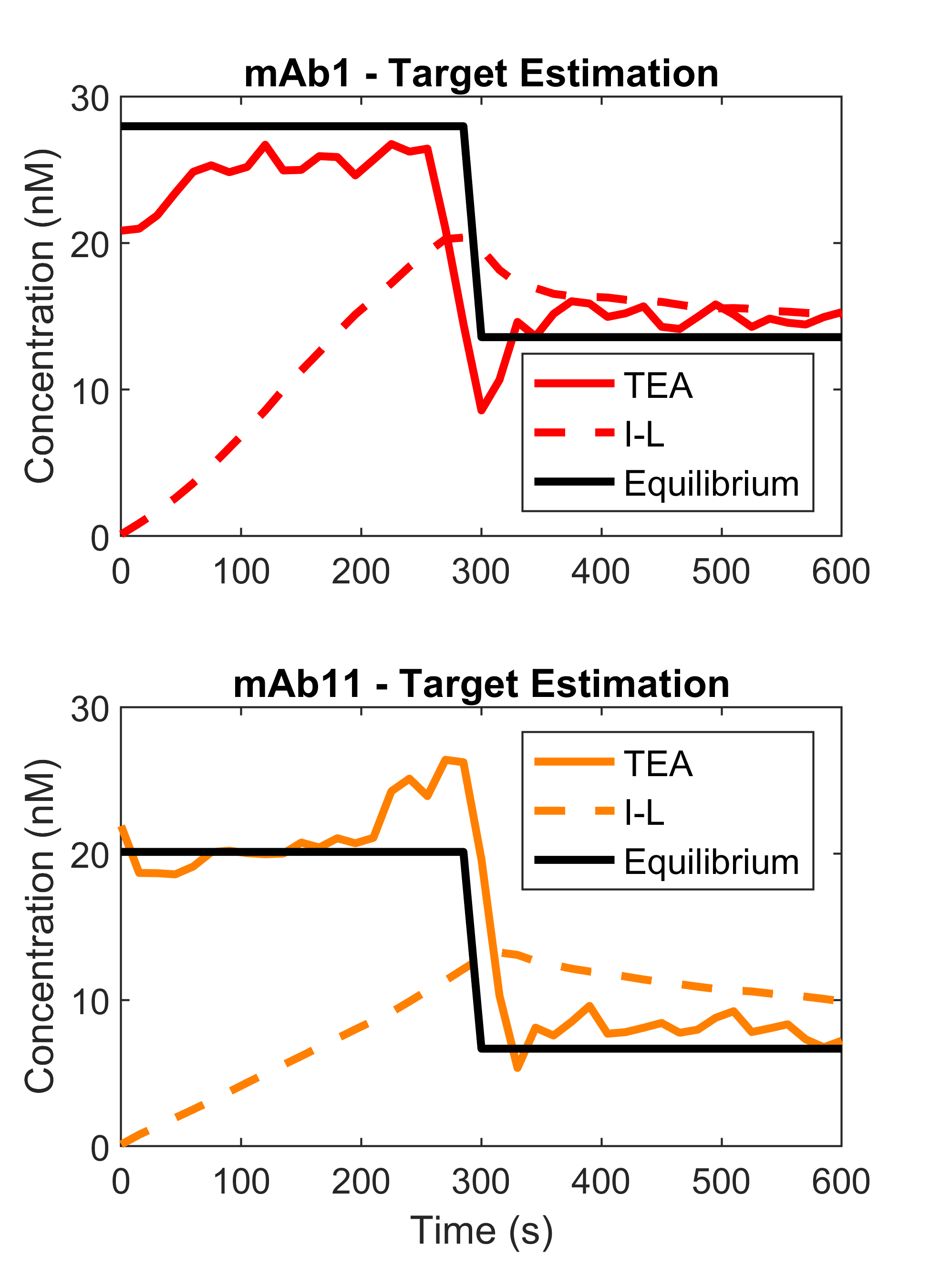

Supplement: Supplementary file 4 — Source Data [file 41467_2022_34778_MOESM4_ESM.zip › Source Data/PaperScripts/OutputFigs/BLIDemoEstimates.png]

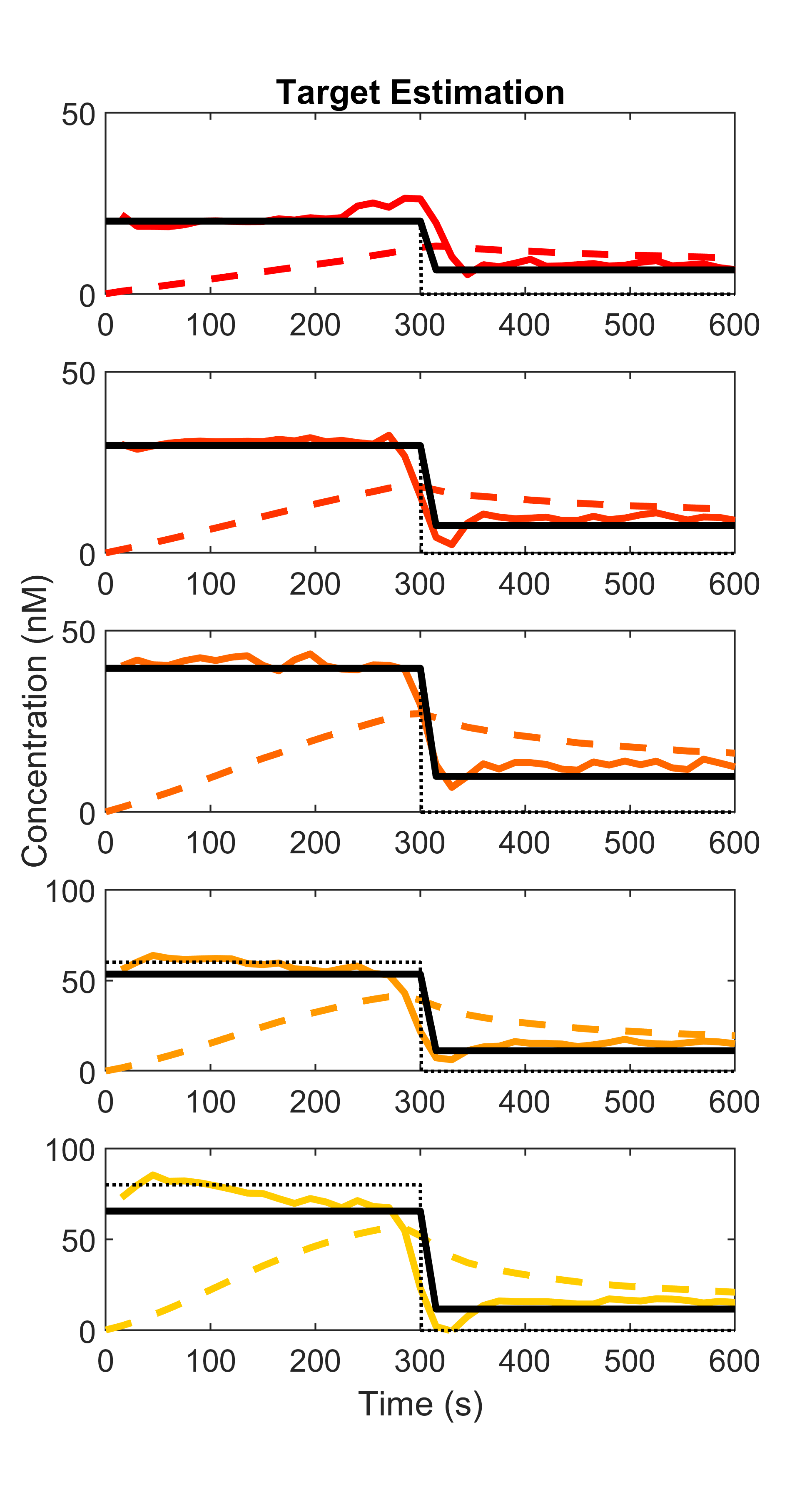

Supplement: Supplementary file 4 — Source Data [file 41467_2022_34778_MOESM4_ESM.zip › Source Data/PaperScripts/OutputFigs/BLIDemoEstimates_allconc.png]

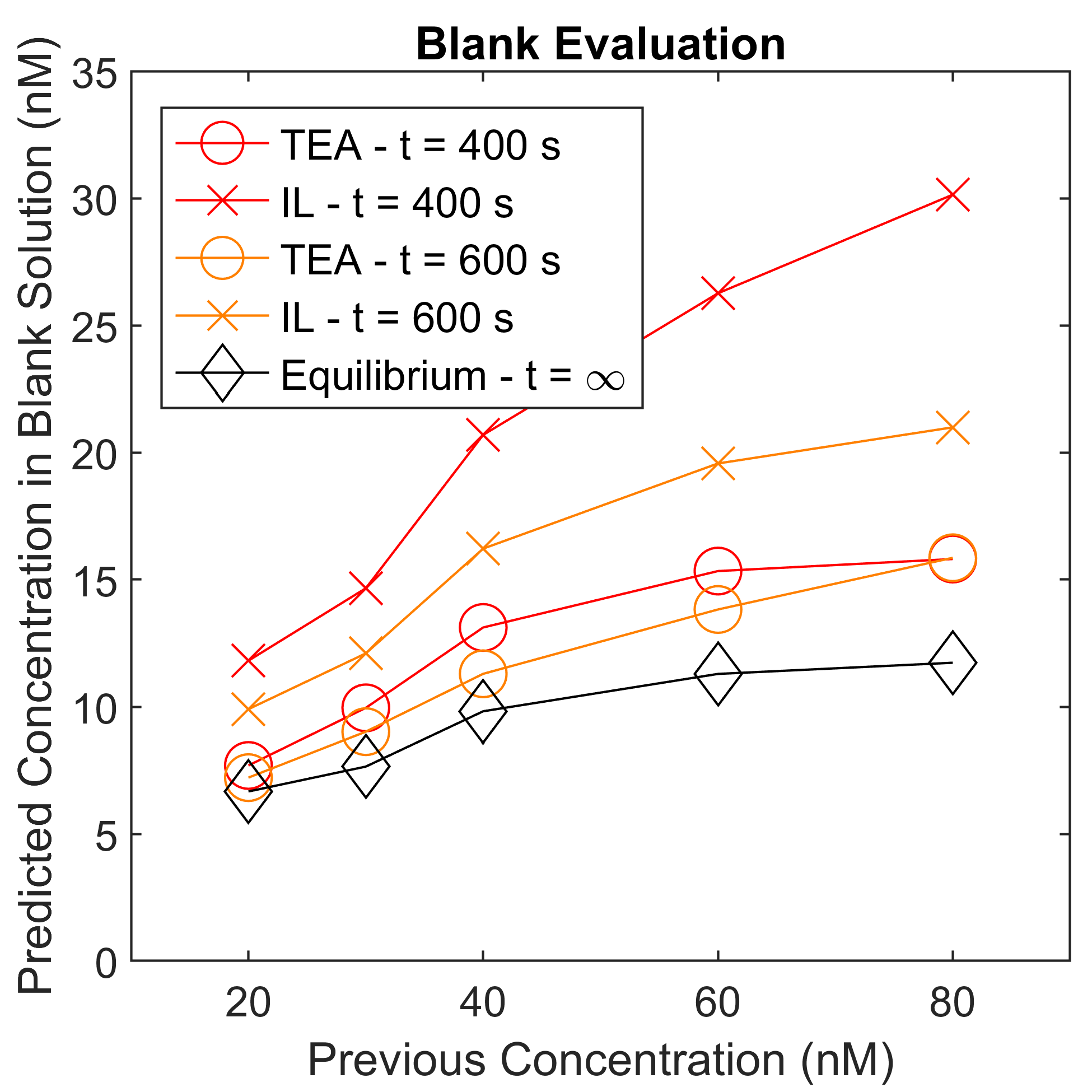

Supplement: Supplementary file 4 — Source Data [file 41467_2022_34778_MOESM4_ESM.zip › Source Data/PaperScripts/OutputFigs/BLIDemoEvalBlank_allconc.png]

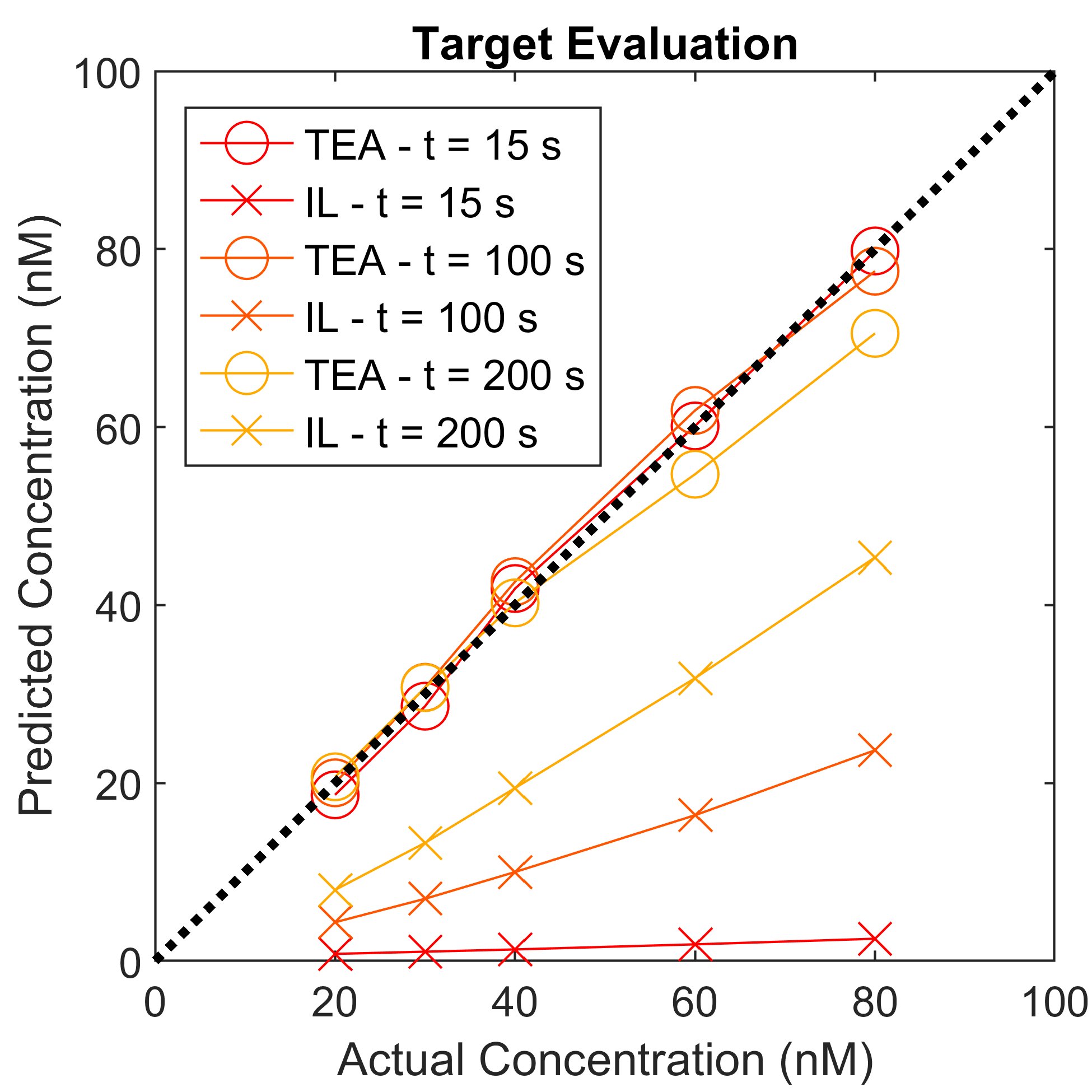

Supplement: Supplementary file 4 — Source Data [file 41467_2022_34778_MOESM4_ESM.zip › Source Data/PaperScripts/OutputFigs/BLIDemoEvalTarget_allconc.png]

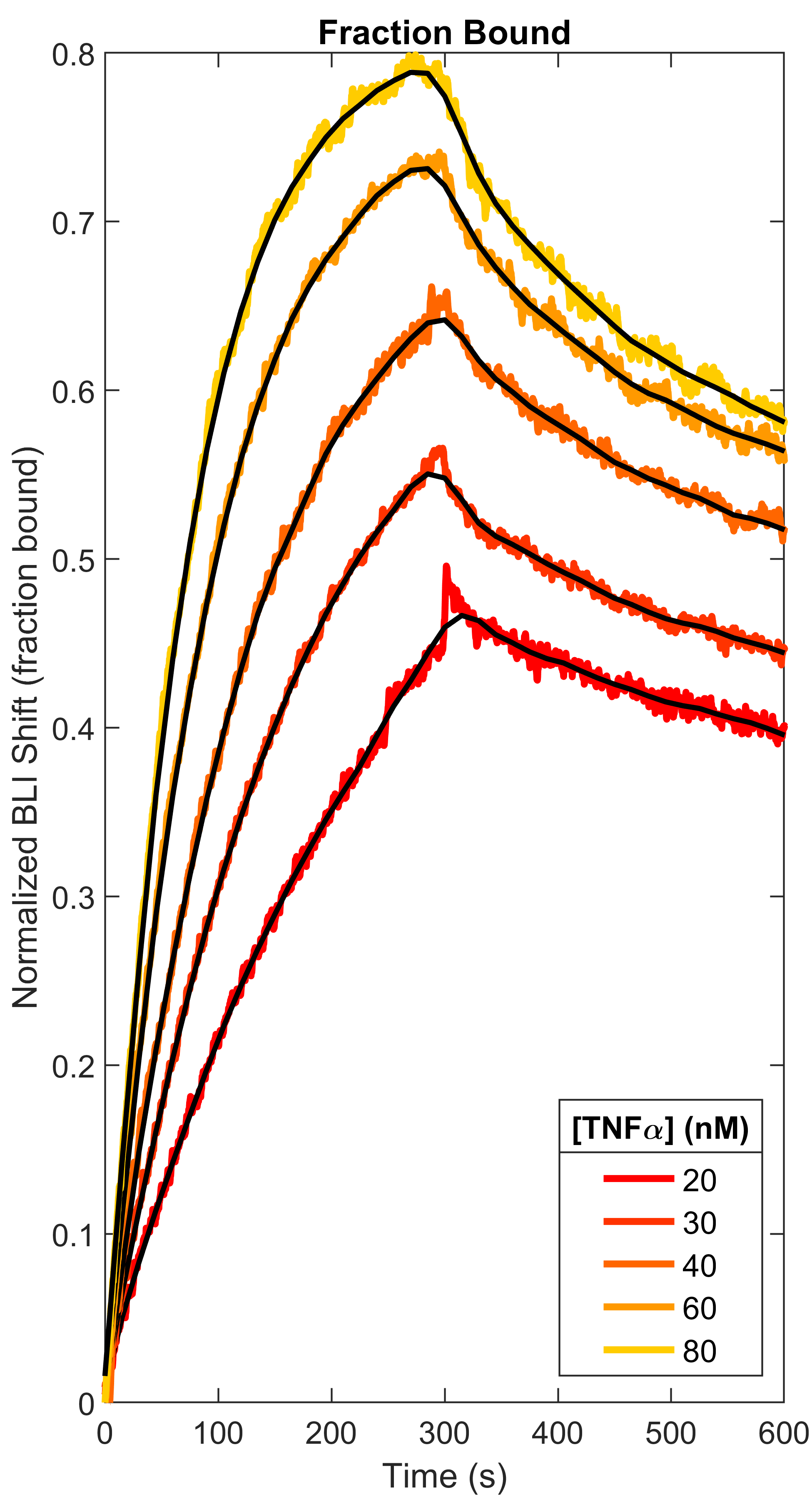

Supplement: Supplementary file 4 — Source Data [file 41467_2022_34778_MOESM4_ESM.zip › Source Data/PaperScripts/OutputFigs/BLIDemoMeasurements_allconc.png]

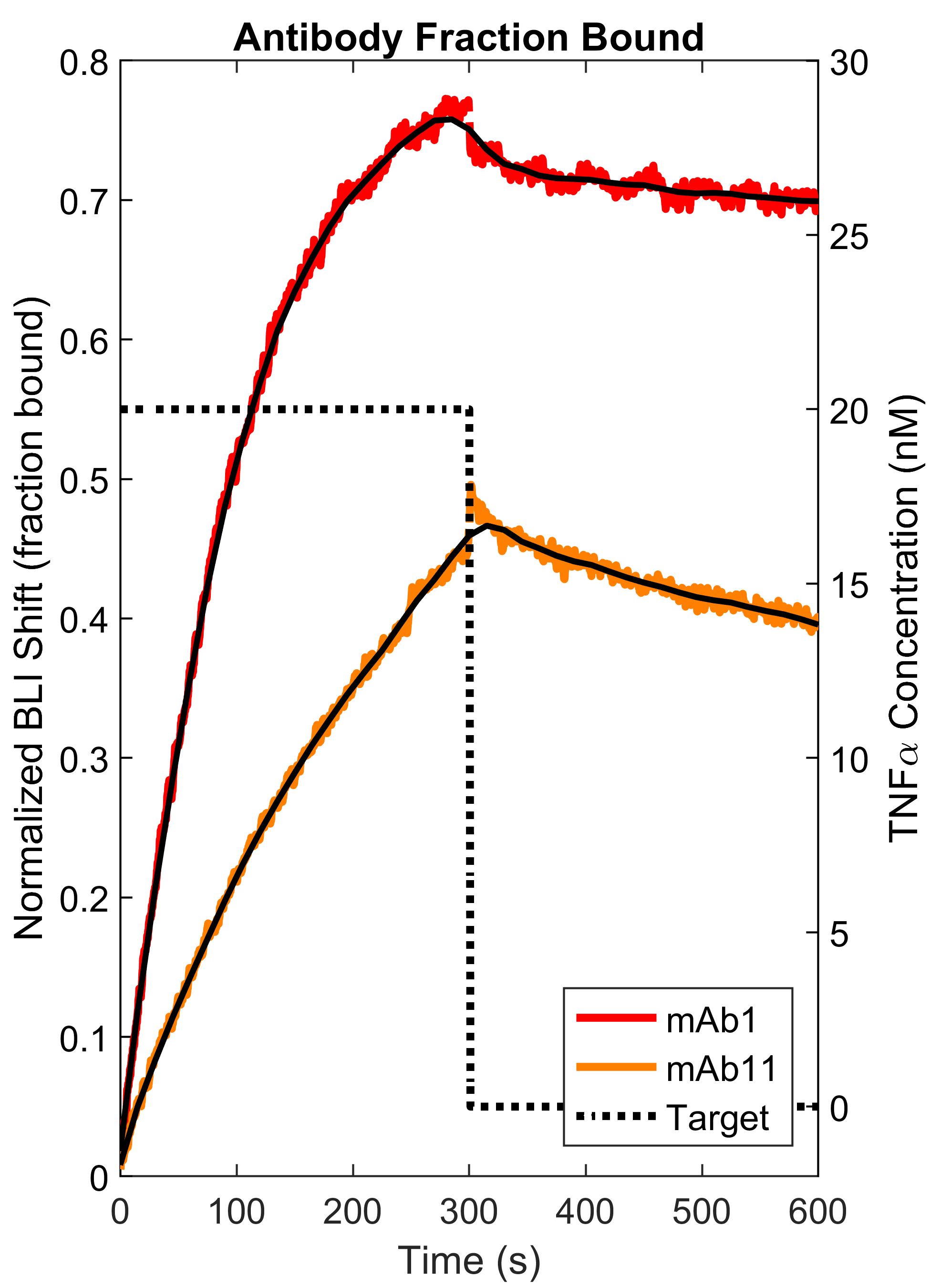

Supplement: Supplementary file 4 — Source Data [file 41467_2022_34778_MOESM4_ESM.zip › Source Data/PaperScripts/OutputFigs/BLIDemoMeasurements_v2.png]

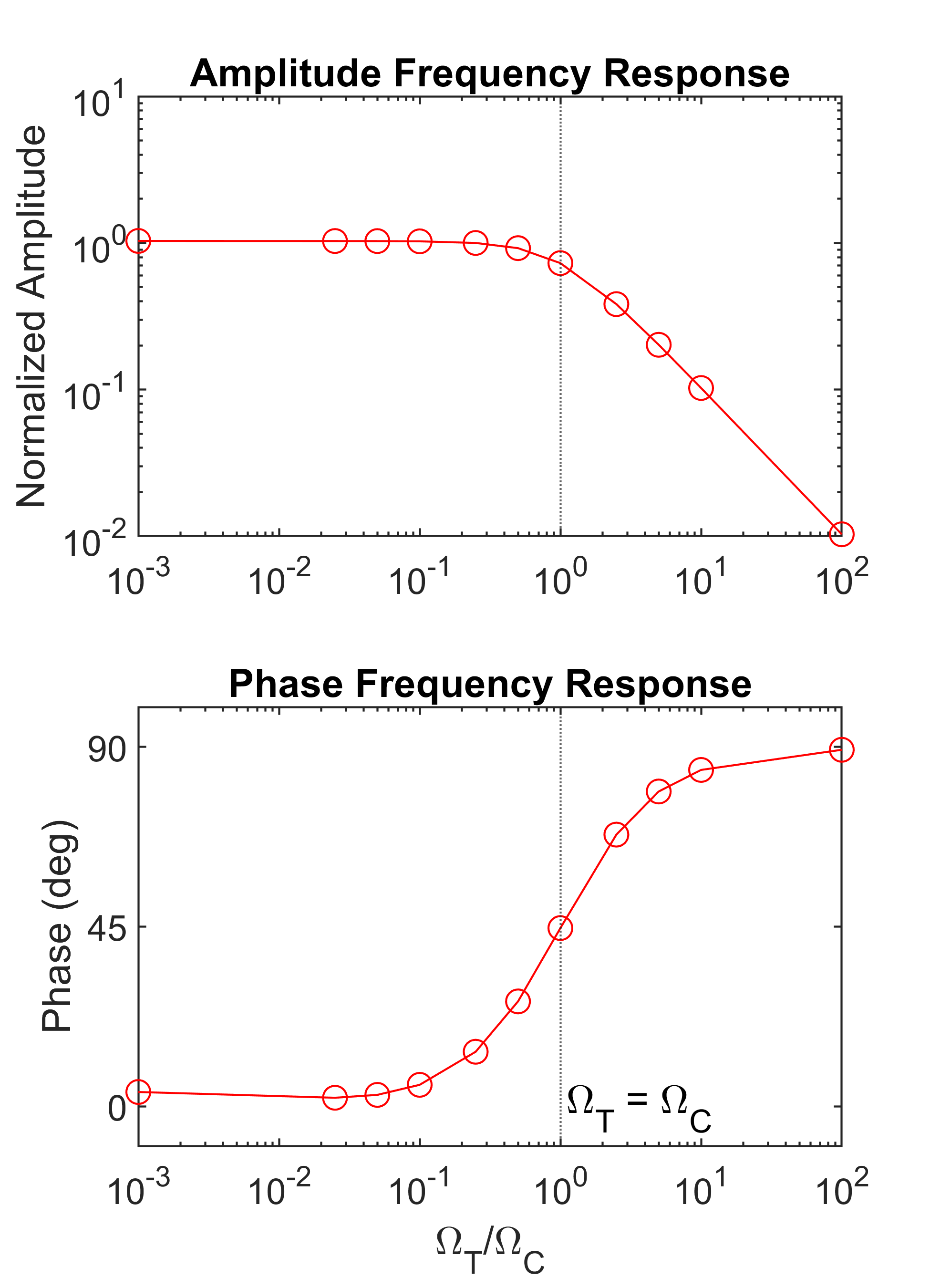

Supplement: Supplementary file 4 — Source Data [file 41467_2022_34778_MOESM4_ESM.zip › Source Data/PaperScripts/OutputFigs/FrequencyResponse.png]

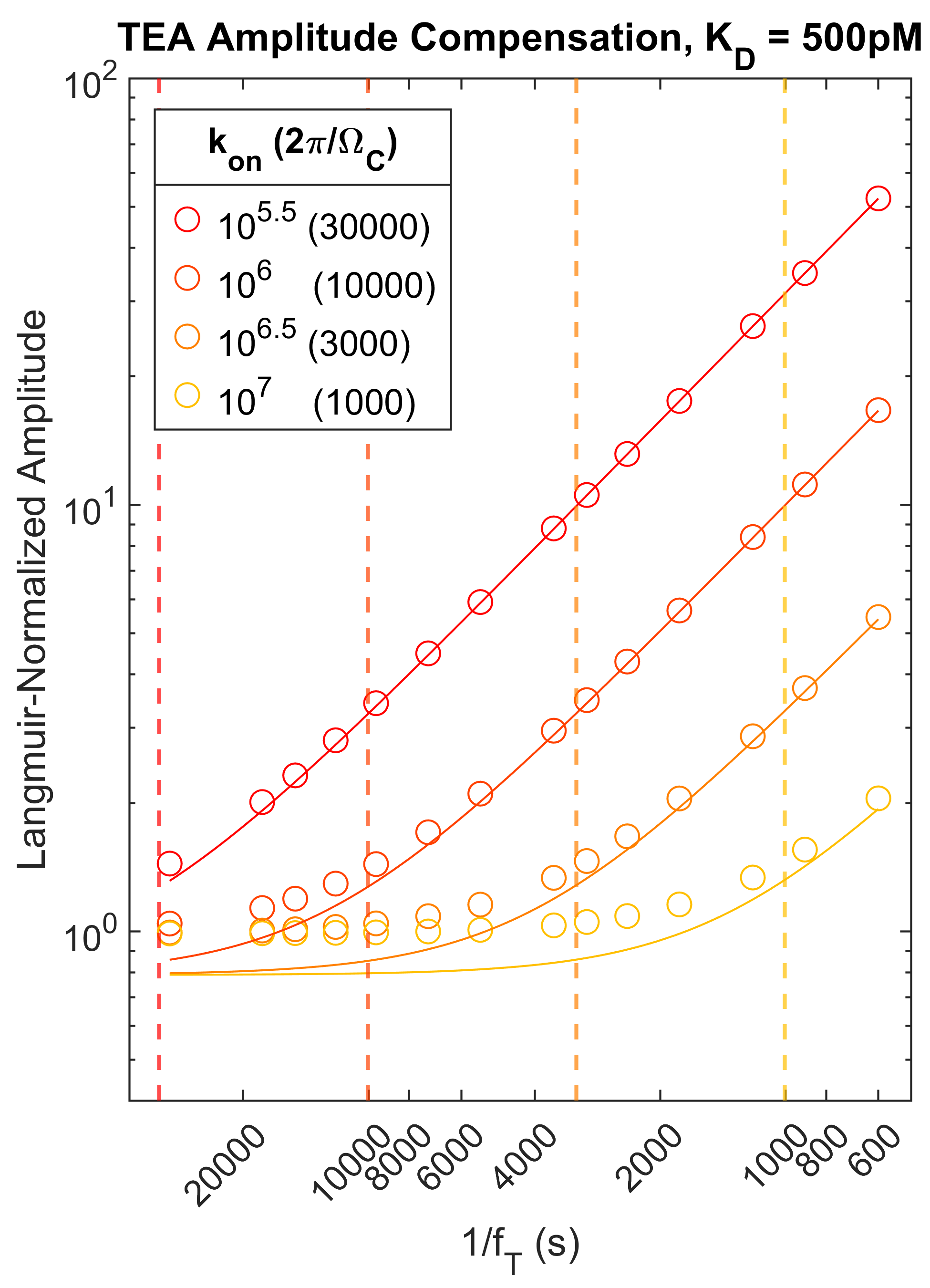

Supplement: Supplementary file 4 — Source Data [file 41467_2022_34778_MOESM4_ESM.zip › Source Data/PaperScripts/OutputFigs/FrequencyResponseTEA.png]

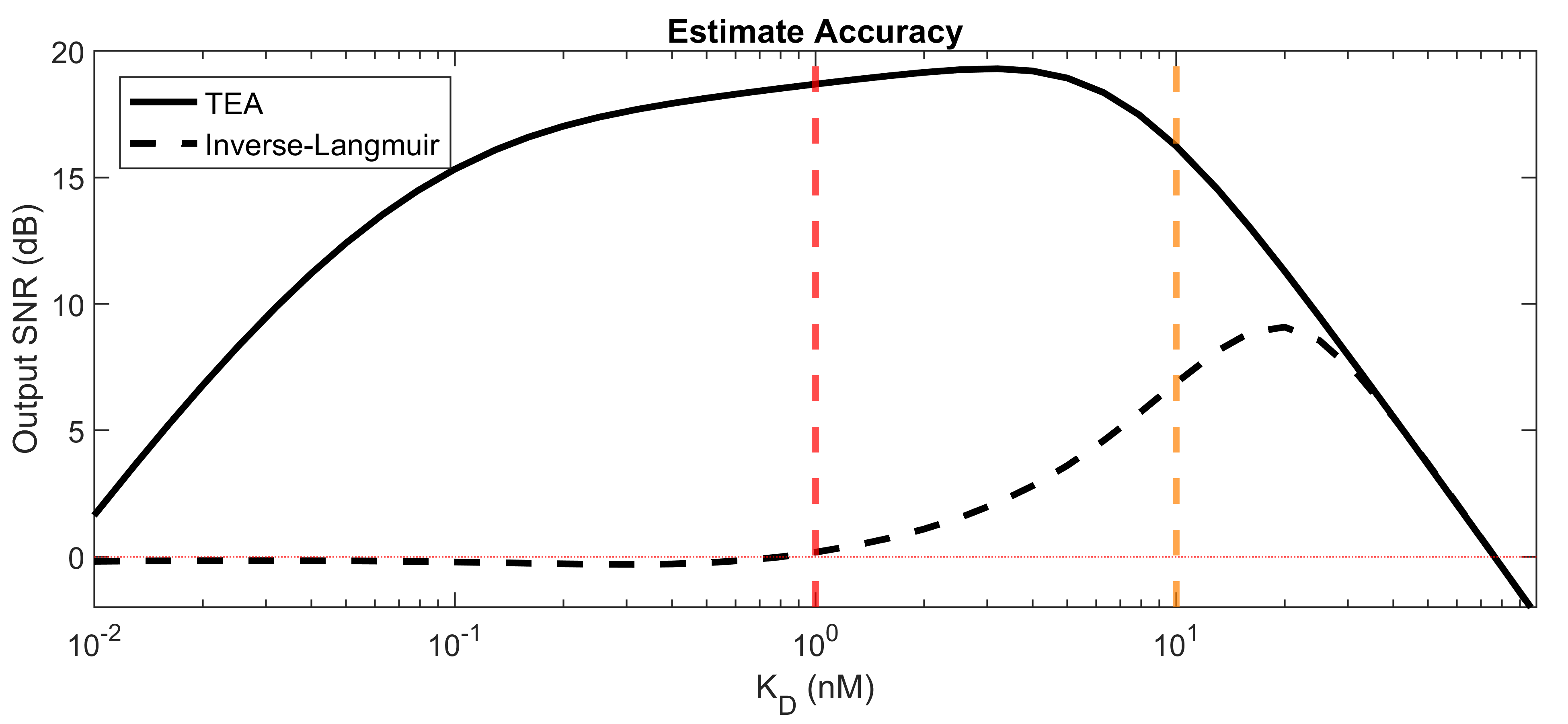

Supplement: Supplementary file 4 — Source Data [file 41467_2022_34778_MOESM4_ESM.zip › Source Data/PaperScripts/OutputFigs/InsulinSNR_v3.png]

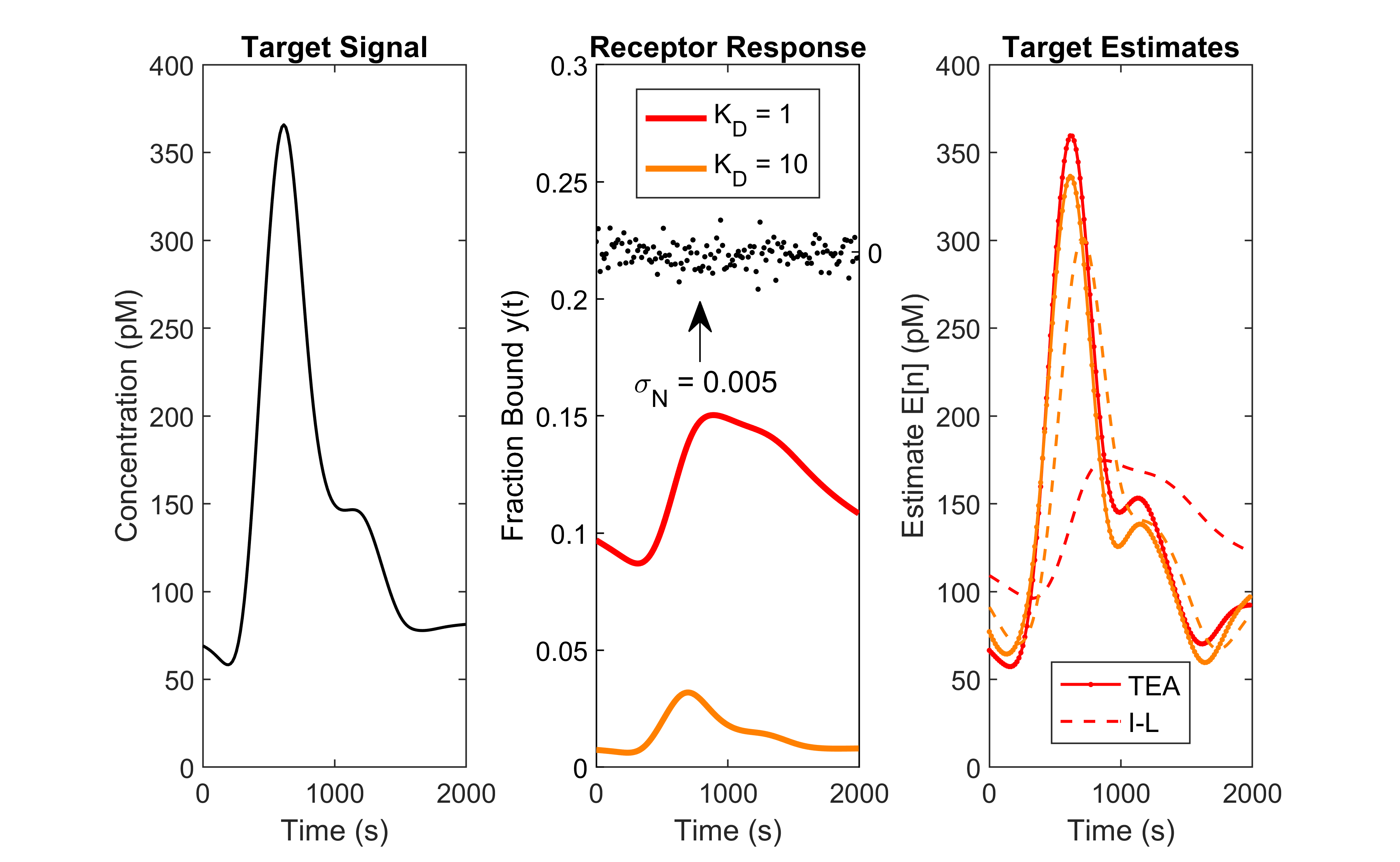

Supplement: Supplementary file 4 — Source Data [file 41467_2022_34778_MOESM4_ESM.zip › Source Data/PaperScripts/OutputFigs/InsulinTimeDomain_v3.png]

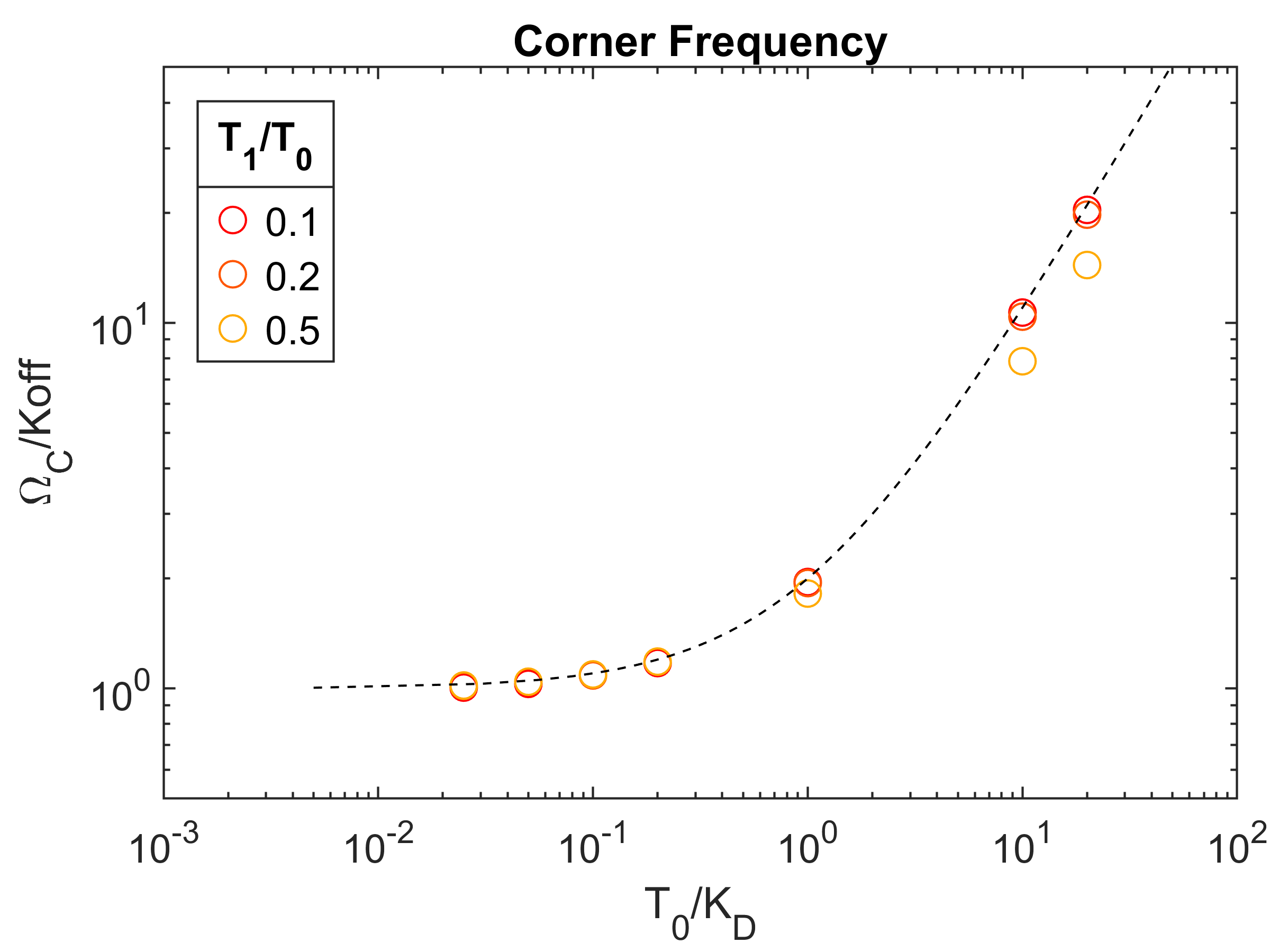

Supplement: Supplementary file 4 — Source Data [file 41467_2022_34778_MOESM4_ESM.zip › Source Data/PaperScripts/OutputFigs/OmegaC_Koff.png]

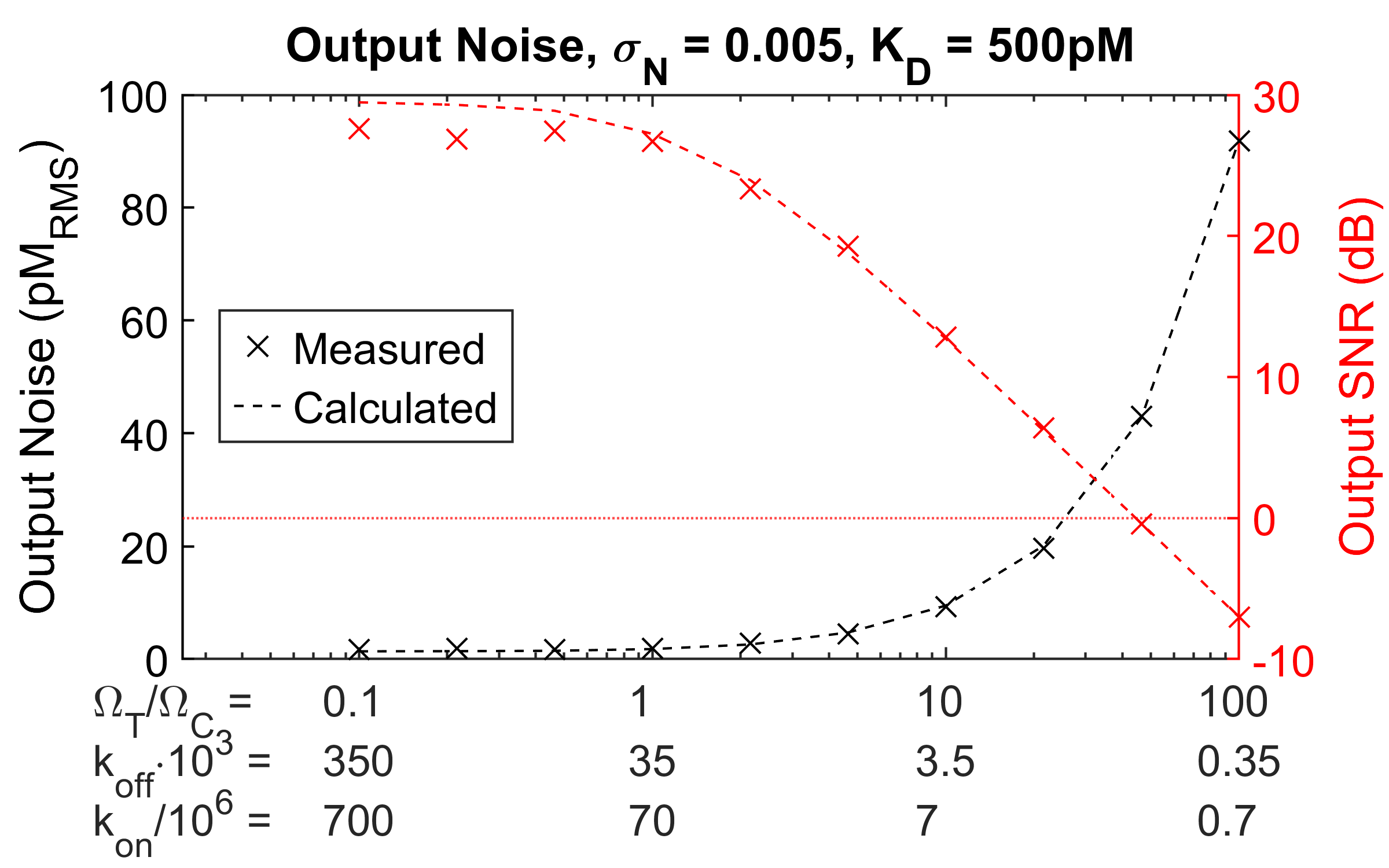

Supplement: Supplementary file 4 — Source Data [file 41467_2022_34778_MOESM4_ESM.zip › Source Data/PaperScripts/OutputFigs/SNRDemoSNR_v2.png]

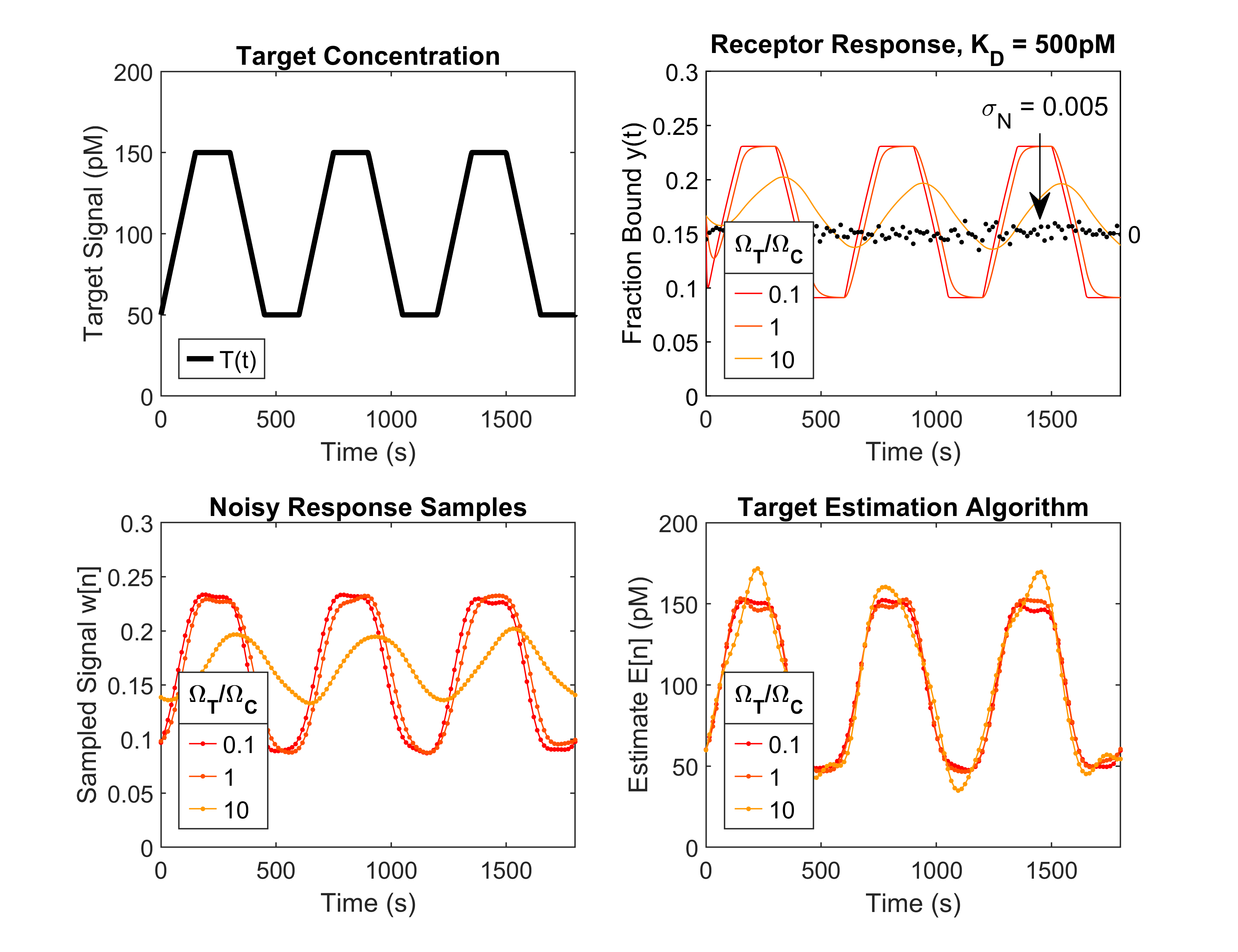

Supplement: Supplementary file 4 — Source Data [file 41467_2022_34778_MOESM4_ESM.zip › Source Data/PaperScripts/OutputFigs/SNRDemoTimeDomain.png]

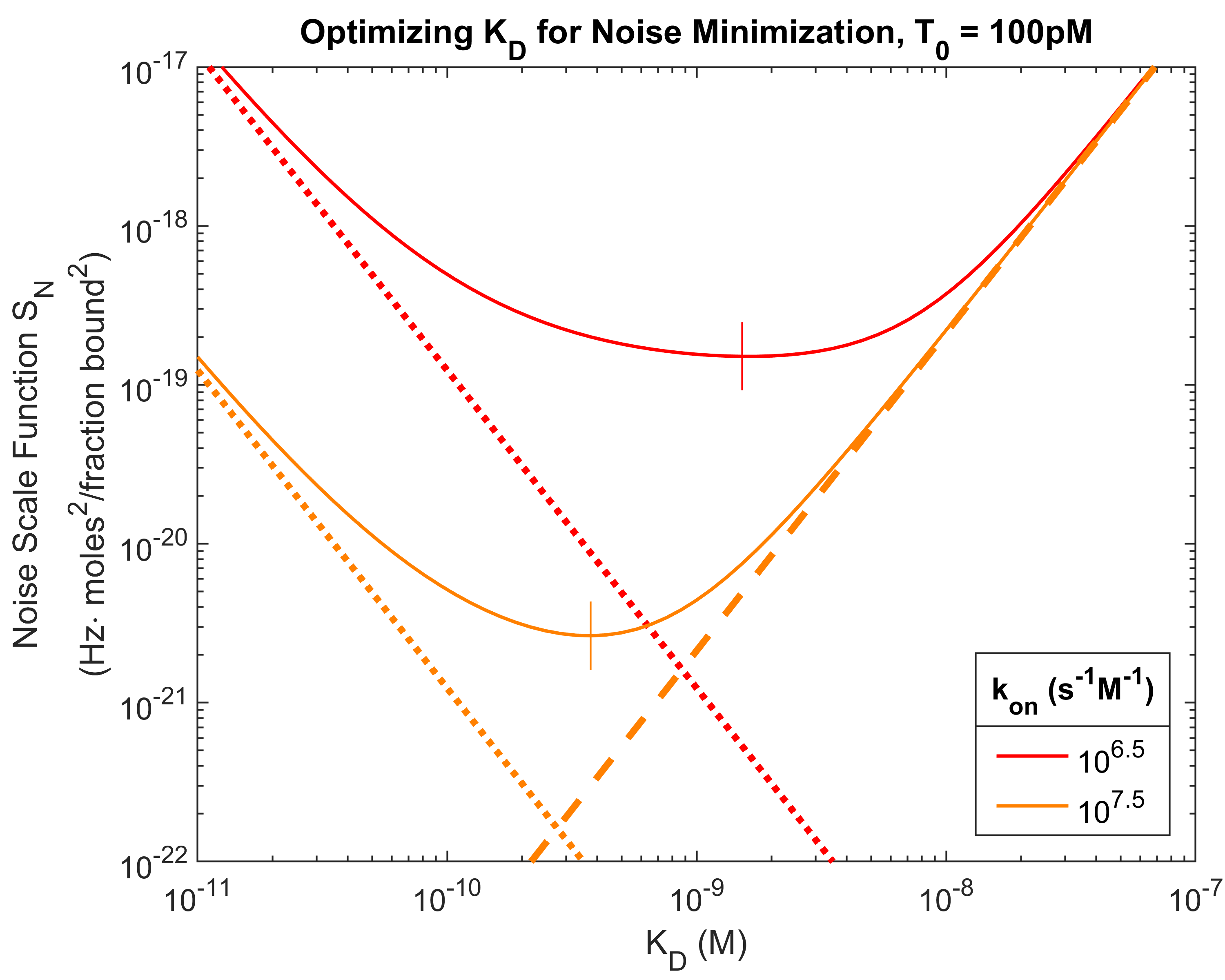

Supplement: Supplementary file 4 — Source Data [file 41467_2022_34778_MOESM4_ESM.zip › Source Data/PaperScripts/OutputFigs/TEAOptimizationKoffV2.png]

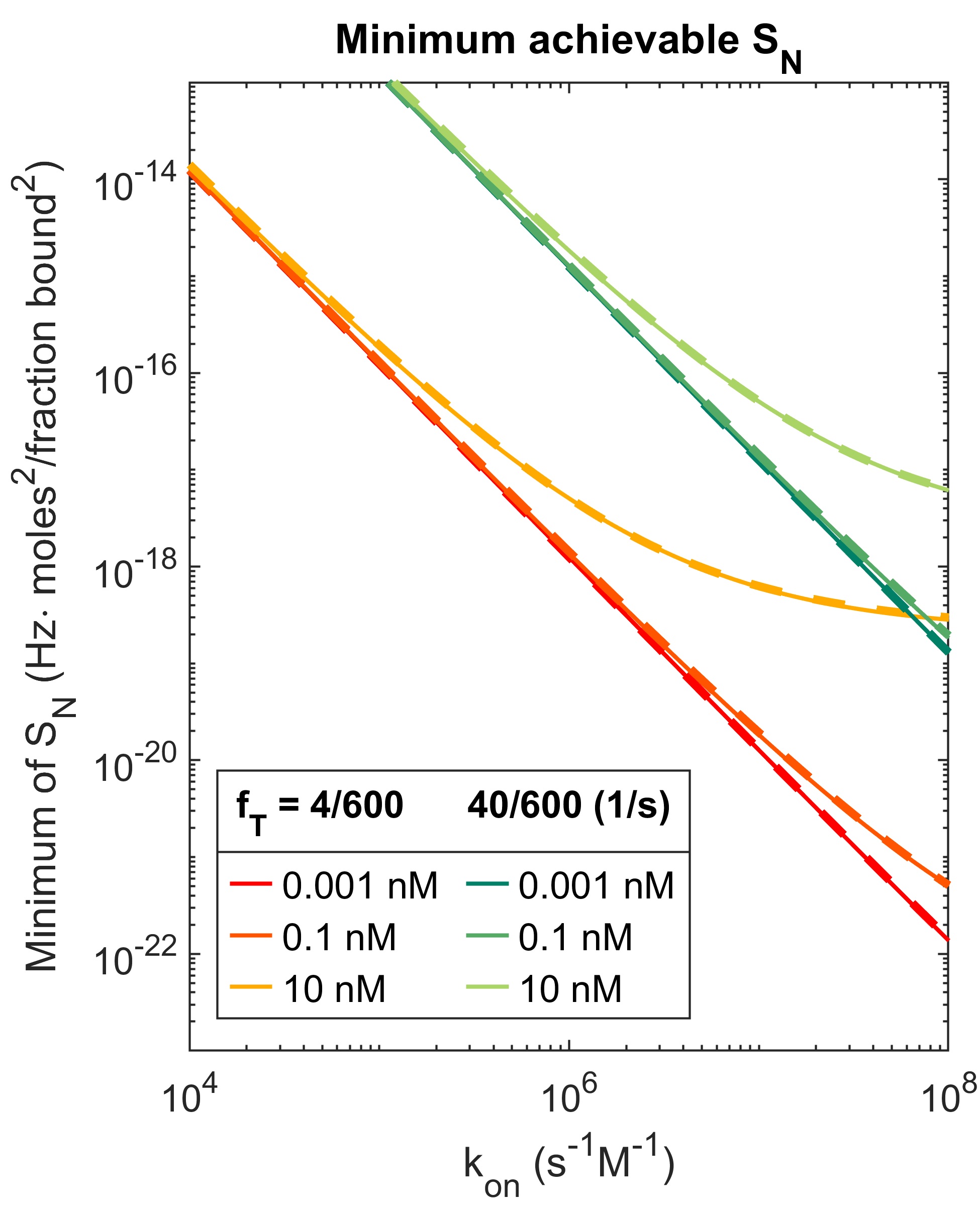

Supplement: Supplementary file 4 — Source Data [file 41467_2022_34778_MOESM4_ESM.zip › Source Data/PaperScripts/OutputFigs/TEAOptimizationSnVsKon.png]

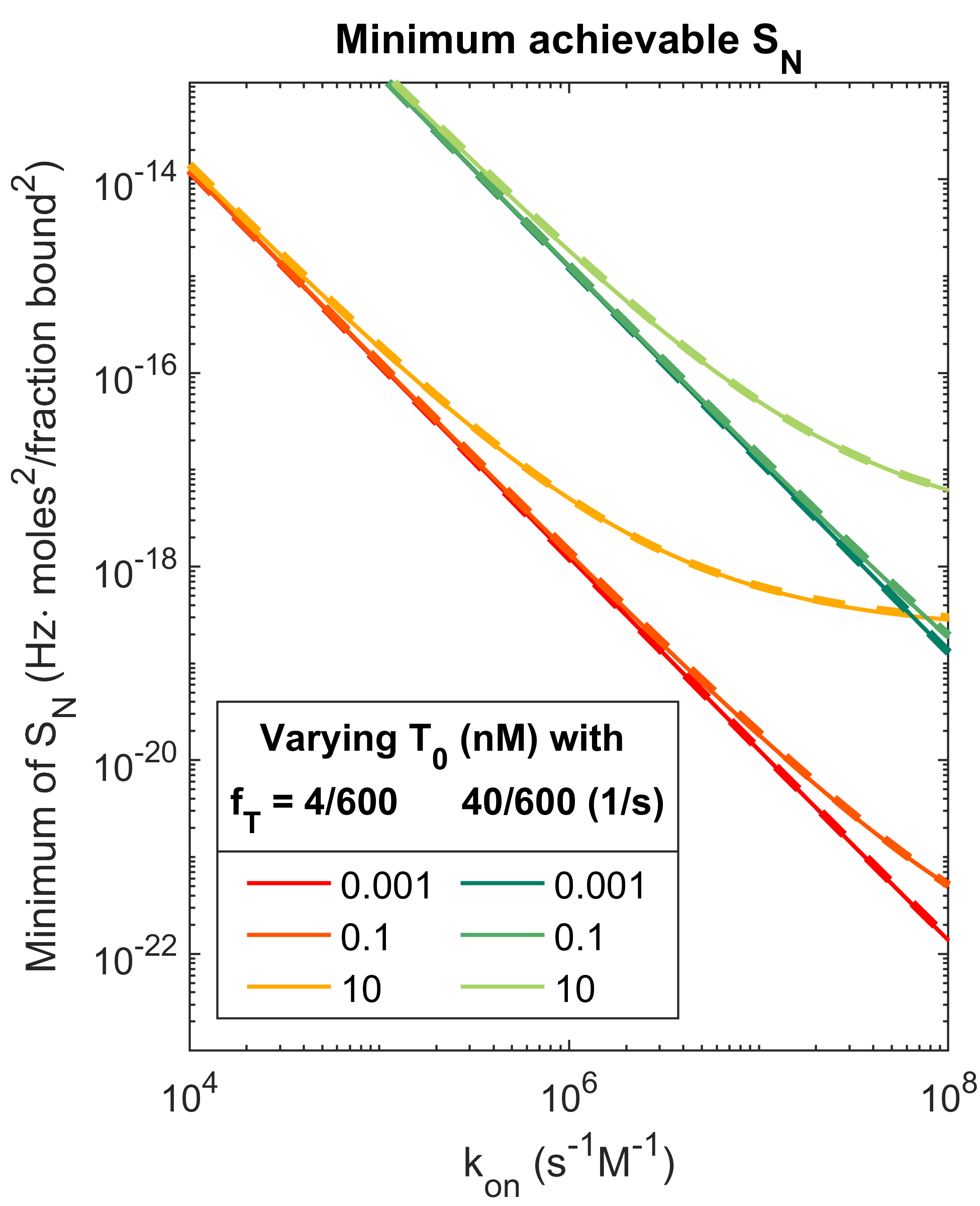

Supplement: Supplementary file 4 — Source Data [file 41467_2022_34778_MOESM4_ESM.zip › Source Data/PaperScripts/OutputFigs/TEAOptimizationSnVsKon_old.png]

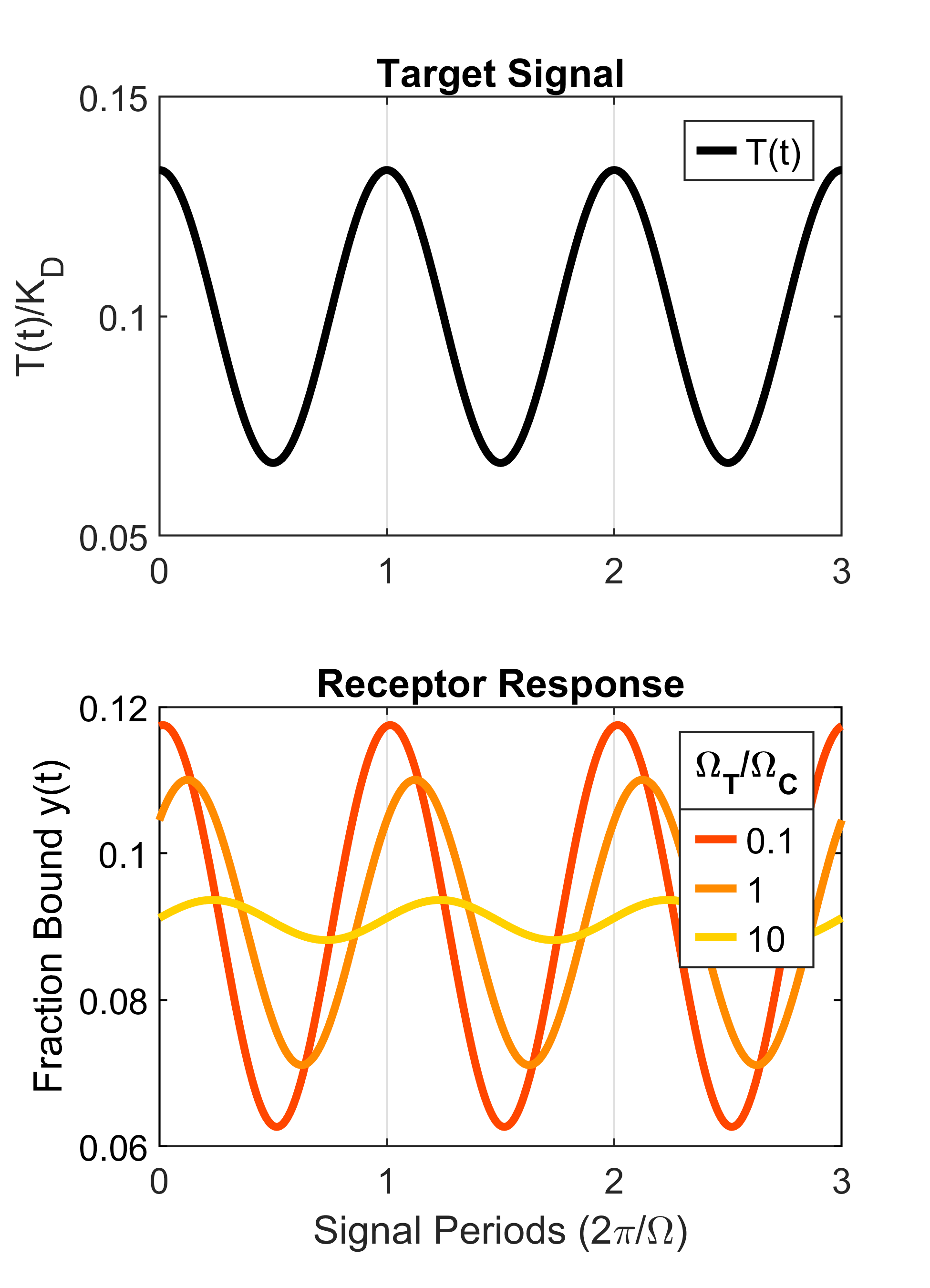

Supplement: Supplementary file 4 — Source Data [file 41467_2022_34778_MOESM4_ESM.zip › Source Data/PaperScripts/OutputFigs/TimeResponse.png]

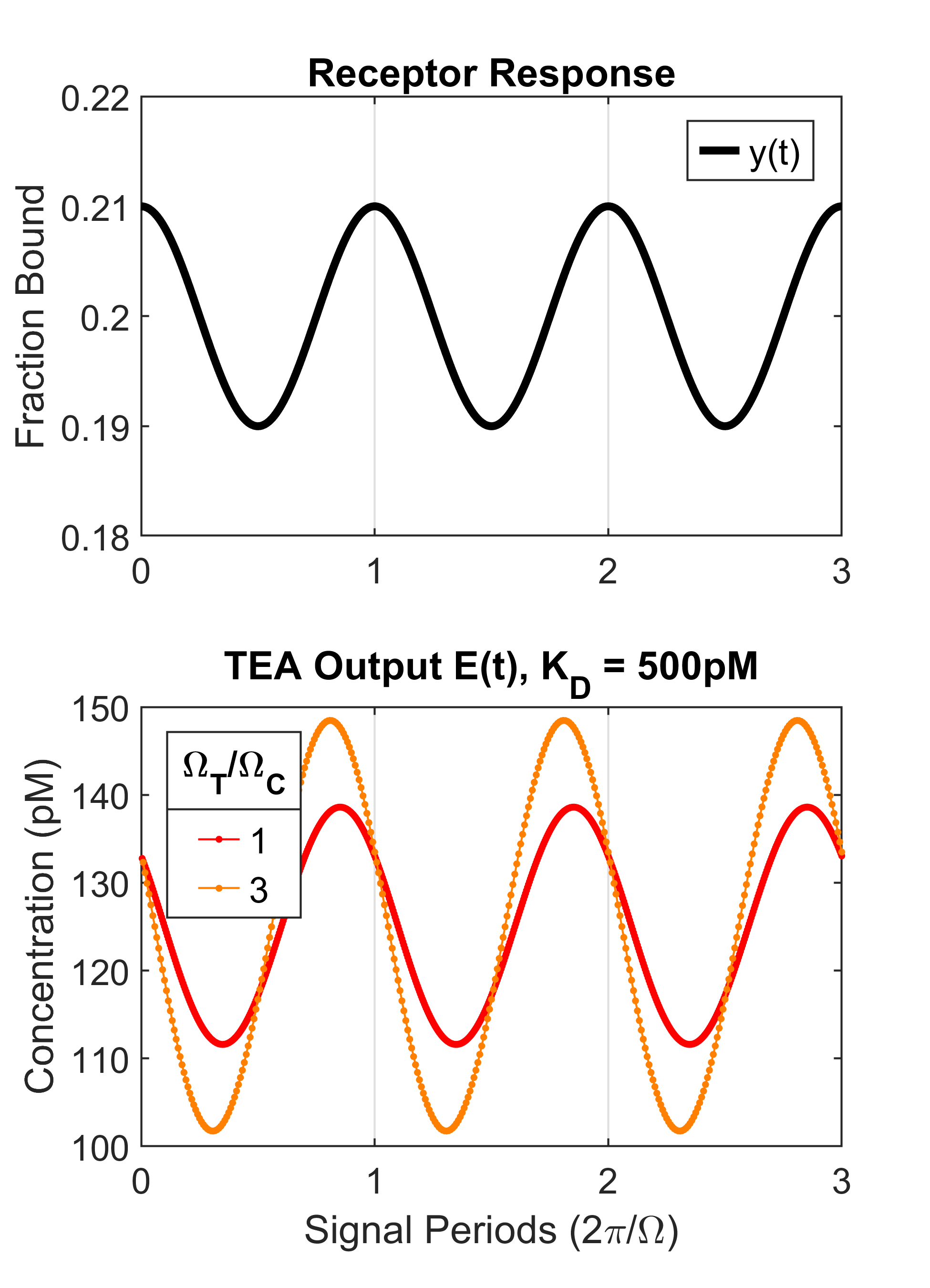

Supplement: Supplementary file 4 — Source Data [file 41467_2022_34778_MOESM4_ESM.zip › Source Data/PaperScripts/OutputFigs/TimeResponseTEA.png]
